# Patch-based dual-domain photon-counting CT data correction with residual-based WGAN-ViT

**DOI:** 10.1088/1361-6560/adaf71

**Published:** 2025-02-06

**Authors:** Bahareh Morovati, Mengzhou Li, Shuo Han, Li Zhou, Dayang Wang, Ge Wang, Hengyong Yu

**Affiliations:** 1 Department of Electrical and Computer Engineering, University of Massachusetts Lowell, Lowell, MA 01854, United States of America; 2 Department of Biomedical Engineering, Rensselaer Polytechnic Institute, Troy, NY 12180, United States of America

**Keywords:** photon-counting CT, transformer, Wasserstein generative adversarial network (WGAN), residual block, TV denoising, guided filtering

## Abstract

*Objective.* x-ray photon-counting detectors have recently gained popularity due to their capabilities in energy discrimination power, noise suppression, and resolution refinement. The latest extremity photon-counting computed tomography (PCCT) scanner leverages these advantages for tissue characterization, material decomposition, beam hardening correction, and metal artifact reduction. However, technical challenges such as charge splitting and pulse pileup can distort the energy spectrum and compromise image quality. Also, there is a clinical need to balance radiation dose and imaging speed for contrast-enhancement and other studies. This paper aims to address these challenges by developing a dual-domain correction approach to enhance PCCT reconstruction quality quantitatively and qualitatively. *Approach.* We propose a novel correction method that operates in both projection and image domains. In the projection domain, we employ a residual-based Wasserstein generative adversarial network to capture local and global features, suppressing pulse pileup, charge splitting, and data noise. This is facilitated with traditional filtering methods in the image domain to enhance signal-to-noise ratio while preserving texture across each energy channel. To address GPU memory constraints, our approach utilizes a patch-based volumetric refinement network. *Main results.* Our dual-domain correction approach demonstrates significant fidelity improvements across both projection and image domains. Experiments on simulated and real datasets reveal that the proposed model effectively suppresses noise and preserves intricate details, outperforming the state-of-the-art methods. *Significance.* This approach highlights the potential of dual-domain PCCT data correction to enhance image quality for clinical applications, showing promise for advancing PCCT image fidelity and applicability in preclinical/clinical environments.

## 1. Introduction

Computed tomography (CT) is a vital imaging technique in clinical practice for assessing anatomical, physiological, and pathological characteristics. To mitigate radiation-induced risks while maintaining high diagnostic image quality, extensive research is ongoing, adhering to the as low as reasonably achievable guideline. This includes fine-tuning scanning parameters like tube voltage, current, pitch, bowtie, and scan time, along with using automatic exposure control. As an emerging CT technology, innovations in photon-counting CT (PCCT) and related algorithms have further reduced radiation doses, benefiting various quantitative CT applications and facilitating novel approaches like simultaneous multi-agent imaging and molecular CT (Söderberg and Gunnarsson 2010, Willemink *et al* 2018, Joyce *et al* 2020, Muhammad *et al* 2022). PCCT, in particular, employs multiple energy windows and much smaller detector sizes, leading to a significantly lower number of x-ray photons in each line integral measurement. Over the past decade, there has been substantial focus on low-dose CT (LDCT) reconstruction, also known as LDCT denoising, which has gained significant momentum following the recent FDA approval of PCCT technology (Hsieh *et al* 2020).

Photon-counting detectors (PCDs) are utilized across various domains, including material science, astronomy, communication, optical imaging, and medical imaging (Robinson *et al* 2006, Verhoeve 2008, Bergamaschi *et al* 2009, Isbaner *et al* 2016, Kalender *et al* 2017). X-ray PCDs have recently gained attention in medical imaging due to their ability to discriminate photons by energy levels. This feature is particularly useful for material decomposition and mitigating issues like beam hardening and metal artifacts. Additionally, PCDs enhance image contrast and reduce radiation dose. The latest x-ray MARS PCCT for extremity imaging (MARS Bioimaging Ltd, Christchurch, New Zealand) capitalizes on these advantages, providing multi-energy high-resolution (HR) imaging for tissue characterization and material decomposition. Moreover, the electronic noise is effectively suppressed from photon and pulse counts since the threshold is set above the electronic noise level but below the pulses generated by incoming photons which results in a higher signal-to-noise ratio (SNR) (Shikhaliev 2009). Additionally, by comparing each pulse to multiple threshold levels, the detector can categorize the incoming photons into various energy bins (Willemink *et al* 2018). However, challenges such as charge splitting and pulse pileup effects can distort the energy spectrum, impacting data quality. Moreover, there is a need to reduce radiation dose and enhance imaging speed for contrast-enhanced and other studies. These phenomena make it difficult for detectors to accurately measure the energy spectrum, ultimately limiting their spatial resolution (Willemink *et al* 2018). Furthermore, there is a strong correlation among different energy bins in PCCT, and reconstructing each energy bin individually can result in noise in the image domain due to the photon count starving problem or excessive quantum noise (Li *et al* 2024). To maintain the reliability of the spectral information produced by PCDs, it is essential to address the impact of these distortions and is particularly crucial since many clinical applications depend on the precise spectral data provided by these detectors. Furthermore, current PCDs that utilize pulse height analysis cannot simultaneously address both charge splitting and pulse pileup by adjusting their parameters, as the two key parameters (pixel size and pulse shaping time) have opposing effects on charge splitting and pulse pileup for a fixed photon flux. This presents a significant challenge to achieve accurate spectral information with current PCD designs. Additionally, most PCDs use a CdTe sensor, which limits the sensitivity for K-edge imaging of iodine, the most common contrast agent in CT (Taguchi *et al* 2018, Holbrook *et al* 2021).

The hardware solutions have been developed to alleviate the spectrum distortions in PCCT data and achieved by the PCD manufacturers after much work (Tkaczyk *et al* 2010, Fu *et al* 2018). Yet, the less expensive algorithms still need to be tested. Numerous studies have designed analytical models to correct pulse pileup effects on the recorded energy spectrum in x-ray PCDs (Taguchi *et al* 2010, Roessl *et al* 2016). In 2010, Taguchi *et al* proposed a model that accounts for factors such as the bipolar shape of the pulse, the distribution function of time intervals between random events, and the input probability density function of photon energies. Monte Carlo simulations demonstrated that the model accurately estimated the recorded spectra for different pulse pileup levels, with coefficients ranging from 5.3% to 10.0% (Taguchi *et al* 2010). In another research by Roessl *et al*, a Fourier approach was employed to predict the expected count rates in a PCD under pulse pileup conditions, considering arbitrary photon flux, detector response function, and pulse shape (Roessl *et al* 2016). Moreover, Treb *et al* demonstrated the feasibility of simultaneously performing photon counting and charge integration in PCDs. They provided the first experimental evidence showing that this approach can correct for pile-up-induced count losses in paralyzable PCDs (2023).

Over the past few years, deep learning has become a leading technology across various scientific and engineering domains, particularly in medical imaging (Morovati *et al* 2023, Wang *et al* 2023, 2024, Han *et al* 2024, Xu *et al* 2024, Zhou *et al* 2024a, 2024b). Based on these analytical foundations, recent years have witnessed deep learning emerging as a dominant force in enhancing CT imaging. This transition is characterized by a shift towards exploring cost-effective correction algorithms through deep learning (Taguchi and Iwanczyk 2013, Touch *et al* 2016, Li *et al* 2020). Touch *et al* proposed a shallow neural network for spectral distortion correction and a joint bilateral filtration denoising method. Nevertheless, they did not succeed in retrieving the photons lost during the charge sharing event and pulse pileup was not addressed (Touch *et al* 2016). To address both pulse pileup and charge splitting correction through supervised deep learning methods, Li *et al* (2020), investigated the use of a single pre-trained convolutional neural network (CNN) as a perceptual loss within a Wasserstein Generative Adversarial Network (WGAN) framework for PCD data correction and employed simulated PCCT data. Their approach significantly improved structural similarity index measurments (SSIM) and peak SNR (PSNR) measurements between the reconstructed images and ground truths. Despite these advancements in modeling PCDs, accurate correction of real PCD data remains challenging due to the complex nature of the physical effects occurring within these detectors. It is worth mentioning that the results after reconstruction of each energy channels are still noisy and need further improvement due to the photon starving effect and non-ideal response of the PCD in each energy channel (Taguchi *et al* 2016). Current image denoising methods can generally be divided into three main categories: preprocessing approaches, iterative reconstruction techniques, and post-processing strategies.

Preprocessing approaches involve manipulating raw data before image reconstruction to mitigate artifacts and noise, including calibration corrections, normalization, and spectral unmixing. Preprocessing denoising techniques suppress noise on projection data, investigating the statistical properties of the noise in the sinogram domain (Wang *et al* 2008). Various methods, such as nonlinear filters (Zhong *et al* 2004) and edge-preserving noise filters (Wang *et al* 2005), have been suggested for CT. These methods effectively minimize streak artifacts and directed noise but can result in an extra loss of spatial resolution if the projection set has the same noise variance. Additionally, this type of denoising may be implemented on the CT scanner’s image reconstruction system, making it scanner-dependent and vendor-reliant, which can increase costs. Xie *et al* formulated a LDCT sinogram preprocessing as a maximum *a posteriori* (MAP) estimation, considering x-ray photon statistics and electronic noise (Xie *et al* 2017). This approach introduced a new prior formulation to better encode piecewise-linear configurations underlying a sinogram. The proposed model more accurately aligns with the statistical essence of sinogram generation and uses an efficient alternating direction method of multipliers algorithm to solve the MAP framework.

Iterative reconstruction techniques have demonstrated success in producing images with low noise levels while preserving crucial details. However, these methods tend to be computationally intensive. Li *et al* (Li *et al* 2024) proposed low-level structural similarity with model-based iterative refinement to solve domain gap issues and training data scarcity. Texture appearance was fine-tuned to align spectral values and enhance structures while suppressing the noise. In recent years, several specialized prior models have been developed for PCCT low-dose reconstruction. These models take into account both intra-channel prior information and inter-channel correlations, demonstrating significant potential for enhancing PCCT reconstruction quality distorted by photon starving artifact. However, these methods demand greater computational resources. Shi *et al* (2024) introduced a tensor neural network for PCCT reconstruction that leverages correlations among different channel images to reduce noise and enhance texture. Initially, spatial texture priors were learned for each channel, which were then combined into a multi-channel network to capture spectral correlations.

Post-processing techniques address the denoising by working directly within the image domain. Post-processing methods can be divided into: deep learning-based approaches and traditional filtering methods. Noise2Sim proposed by Niu *et al* was a self-supervised deep denoising approach that addressed the limitations of current methods in processing correlated noises in PCCT images. Unlike the traditional supervised methods, which required paired clean or noisy samples, Noise2Sim operated in a non-local and nonlinear fashion to suppress both independent and correlated noises (2022). Recently, Hein *et al* (2024) introduced posterior sampling Poisson flow generative models (PPFM) for LDCT and PCCT denoising to achieve high image quality. By updating the PFGM++ training and sampling processes, a conditional generator was learned to maintain a direct trajectory between the noise and posterior distributions. However, deep learning and particularly generative models are computationally expensive and time consuming. In light of the computational demands and complexity associated with advanced deep learning models, the traditional denoising methods remain highly relevant. Given that noise is a statistical fluctuation dictated by quantum mechanics, denoising is typically accomplished through mean or averaging operations. Common techniques include local averaging methods such as Gaussian smoothing (Lindenbaum *et al* 1994), neighborhood filtering (Smith and Brady 1997), wavelet transform filtering (Rabbani *et al* 2009), total variation (TV) denoising (Vogel and Oman 1996, Chen *et al* 2010), and guided filtering (GF) (He *et al* 2012). These methods are well-regarded for their simplicity and effectiveness in different scenarios. Considering the critical importance of edge preservation and texture details for diagnostic purposes in CT images, researchers have extensively leveraged traditional denoising methods in the image domain (Borsdorf *et al* 2008, Diwakar and Kumar 2018, Omer *et al* 2018). Among these techniques, TV minimization is particularly beneficial. It effectively suppresses noise and artifacts while preserving and enhancing texture details, making it highly suitable for medical imaging applications (Chen *et al* 2010). Furthermore, the traditional methods such as GF offer computational efficiency and the capability to handle high-dimensional data, which is crucial in clinical settings where quick and reliable results are necessary. Traditional denoising methods also come with the advantage of being well-understood and relatively easy to implement, making them a valuable complement to more sophisticated deep learning approaches. They provide a solid foundation for noise reduction that can be further refined with more advanced techniques, thus ensuring that critical diagnostic details are retained.

In this study, we build upon the work proposed by Li *et al* (2020), by investigating PCCT data correction in two domains. Our approach involves a structural modification of WGAN to capture both global and local features in the projection data through residual blocks. This is followed by a TV denoising and GF in image domain to preserve important features, such as edges, while suppressing quantum noise. We propose to use a single network as the generator to directly map distorted, noisy projections to high-quality ground-truth projections for noise-reduction. Additionally, we integrate residual blocks into the discriminator structure to leverage both global and local features effectively. As demonstrated in our previous work (Morovati *et al* 2024), we utilize the vision transformer (ViT) as the perceptual loss network to enhance global contextual understanding of PCD data correction and capture long range feature dependencies. This approach is beneficial for extracting more informative and textured features (Vaswani *et al* 2017, Dosovitskiy *et al* 2020, Wang *et al* 2024). The goal of this investigation is to improve the accuracy and reliability of PCD data correction through residual-based WGAN-ViT (R-WGAN-ViT) combined with the traditional denoising methods to suppress spectral distortions in projection domain and quantum noise in image domain. This paper makes a substantial advancement over our previous work (Morovati *et al* 2024). Our findings reveal outstanding performance on both simulated PCCT phantom data and real PCCT data, showcasing the potential of our approach to revolutionize PCCT imaging and practical applications. It is noteworthy to mention that our primary objective is to address the unique challenges of PCCT, including charge splitting, pulse pileup, and Poisson noise, through a robust dual-domain correction framework. By training our model on simulated phantom data and testing it on real chicken leg phantom data, we demonstrate its capability to generalize effectively. These results suggest that our model holds significant potential for extremity imaging (*e.g.* hands, feet, legs, arms) using a MARS extremity clinical PCCT scanner (MARS Bioimaging Ltd, Christchurch, New Zealand), where noise reduction and spectral fidelity are critical.

The remainder of this paper is organized as follows: section 2 outlines the motivation and problem formulation, detailing the proposed model for PCD data correction and image reconstruction approach. Section 3 presents the experiments and results, including a comparative analysis of the obtained outcomes and the ablation study results. Finally, section 4 provides a discussion of the findings, concludes the study, and outlines potential directions for future research.

## 2. Materials and methods

### 2.1. Overview

PCCT represents a significant advancement over the traditional CT by offering enhanced contrast resolution and material decomposition based on its spectral properties. However, these benefits are accompanied by challenges such as pulse pileup and charge splitting in the projection domain, which are prevalent in PCDs. Additionally, quantum noise or count starving effects can occur in each reconstructed energy bin in PCCT. While different deep learning-based methods have been proposed to address quantum noise artifacts, there has been considerably less methods focusing on correcting pulse pileup and charge splitting simultaneously. Moreover, it is crucial to account for both global and local features of PCCT data in projection domain, and ensure the preservation of edges and texture details while effectively suppressing noise in image domain.

**Figure 1.**
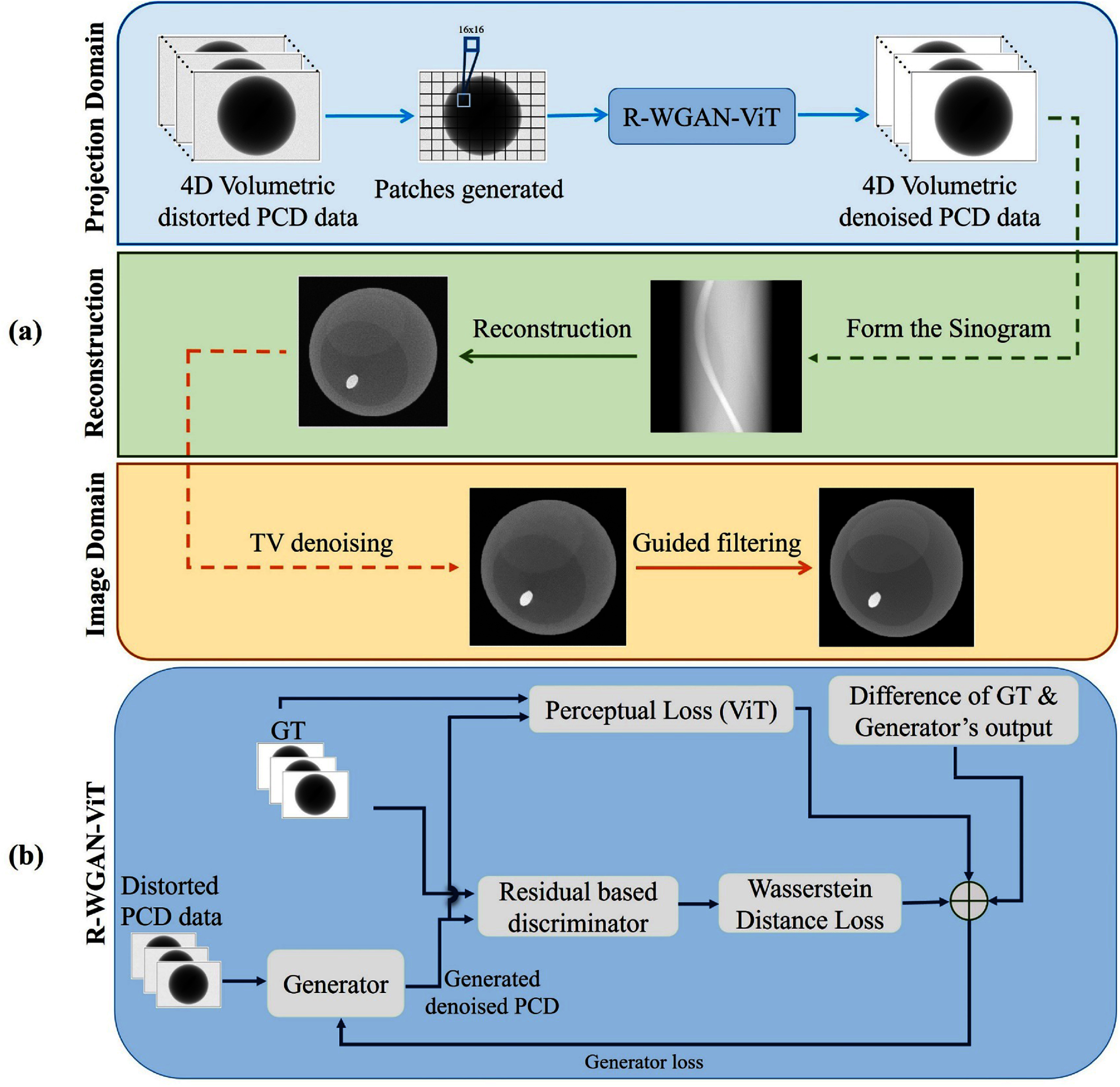
Overview of the proposed PCCT data correction in dual domain. (a) Three main components of our approach with R-WGAN-ViT and traditional denoising, (b) architecture of the R-WGAN-ViT network.

To tackle the aforementioned challenges, we propose a dual-domain approach by utilizing a Residual WGAN with ViTs (R-WGAN-ViT), combined with the traditional filtering methods. This method transforms distorted projection data, affected by pulse pileup and charge splitting, into projections with substantially reduced noise, which are then used for reconstruction by employing filtered backprojection (FBP) and the simultaneous algebraic reconstruction technique (SART). Finally, the traditional denoising techniques are applied in the image domain to suppress noise while preserving essential texture details and edges. Our approach corrects distortions in both the projection and image domains. An overview of our method is presented in figure 1(a), comprising three main components:

1. **Pulse pileup and charge splitting correction in the projection domain:** using the R-WGAN-ViT, we integrate residual blocks into the discriminator, enabling the network to learn residual functions. We leverage the ViT as the perceptual loss to capture long-range feature dependencies and global features.
2. **Reconstruction of denoised PCCT sinogram:** the second stage involves reconstruction from the denoised PCCT sinogram using the FBP and SART methods, considering parallel beam and cone beam circular scanning geometry for simulated phantom data and real PCCT data, respectively.
3. **TV denoising and GF in the image domain:** the final stage applies TV denoising and GF in the image domain to mitigate count starving effects or quantum noise while preserving critical information.

The details of our approach, including the structure and design of the R-WGAN-ViT and subsequent steps, are elaborated in the following sections.

### 2.2. Pulse pileup and charge splitting correction in the projection domain

We use the R-WGAN-ViT for PCCT projection correction due to its ability to effectively address distortions such as pulse pileup and charge splitting, which are common challenges in PCCT. The R-WGAN-ViT framework leverages the strengths of residual networks and ViT to capture both local and global features in the projection data. The residual blocks in the network help in learning residual functions, improving the model’s capacity to correct detailed distortions. The ViT is used as a perceptual loss to consider long-range feature dependencies and global context, ensuring a comprehensive understanding of the data’s structural information which is calculated between the generator’s output and the ground truth. This combination enhances the accuracy of the projection correction, ultimately leading to higher quality and more reliable PCCT images. The general architecture of the proposed framework for R-WGAN-ViT is depicted in figure 1(b). The details of R-WGAN-ViT are outlined in the following subsections.

#### 2.2.1. WGAN

**Figure 2.**
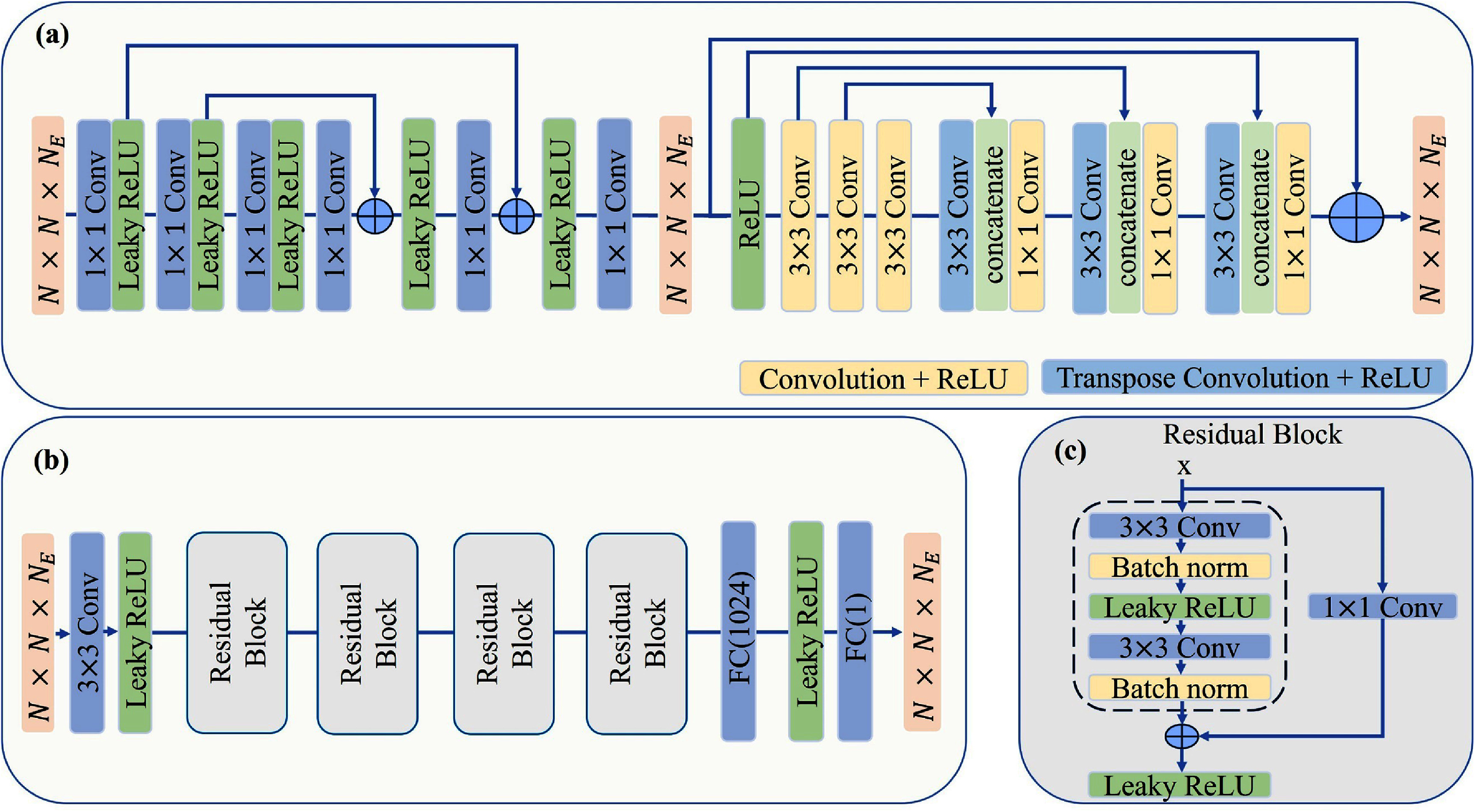
Architecture of the generator and discriminator, where $N$ and ${N_{\text{E}}}$ stand for patch size and number of energy bins, respectively. (a) Generator, (b) residual-based discriminator, and (c) detailed structure of the residual block.

Generative Adversarial Networks (GANs) have emerged as a powerful deep learning technique for generating realistic data, such as images, audio, and natural language, that closely resemble real data. GANs consist of two neural networks: a generator and a discriminator. The generator learns to produce samples from a random noise distribution (often sampled from a Gaussian or uniform distribution). This noise serves as the starting point, allowing the generator to explore different data representations. Through learned transformations, the generator maps this noise to the desired data distribution. Meanwhile, the discriminator learns to distinguish between real data and the generated samples. These networks are trained adversarially. The generator aims to create samples that can fool the discriminator, while the discriminator strives to accurately identify real versus generated data. This adversarial training process enhances the generator’s ability to produce more realistic data and improves the discriminator’s accuracy in identifying generated samples (Goodfellow *et al* 2014, Arjovsky *et al* 2017). In the context of PCDs, ‘realistic data’ refers to projection images that replicate the characteristics of actual PCD data (ground truth), including the appropriate spectral distribution and minimized noise from charge splitting and pulse pileup. As an advancement over the conventional GANs, Wasserstein GANs utilize the Wasserstein distance instead of the Jensen-Shannon divergence to measure the difference between data distributions. By incorporating the Wasserstein distance in the loss function, WGANs can generate higher-quality data and mitigate some of the training difficulties encountered with the traditional GANs (Gulrajani *et al* 2017). The adversarial loss function in WGANs is formulated as follows:

\begin{equation*}\mathop {\min }\limits_D \mathop {\max }\limits_G {L_{\text{WGAN}}}\left(D,G\right) = - {E_P}\left[D\left(p\right)\right] + {E_m}\left[D\left(G\left(m\right)\right)\right] + \lambda {E_{\hat p}}\left[{\left({\left\| {\nabla \hat pD\left(\hat p\right)} \right\|_2} - 1\right)^2}\right],\end{equation*}

where $G,D$, and $E$ stand for the generator, discriminator, and estimated value, respectively, while $p$ and $m$ respectively represent the ideal projection data and the degraded projection measurement. Here, ‘ideal projection data’ refers to a target projection—that our model aims to approximate in the output by learning to correct the distortions in the degraded input data. The first and second terms in equation (1) estimate the Wasserstein distance, and the third term acts as a gradient penalty for network regularization. This penalty serves as an alternative to directly restrict weight values and effectively enforces a Lipschitz constraint on the discriminator, enhancing stability during training. The symbol $\hat p$ denotes points uniformly sampled along a straight line connecting the generated data $G(m)$ and real data point $p$. The parameter $\lambda $ adjusts the influence of the penalty term, allowing the WGAN to fine-tune its effect on the overall loss function. In previous studies, a WGAN was used to develop a mapping function $G$ that transforms distorted projection measurements $m \in {\mathbb{R}^{N \times N \times {N_{\text{E}}}}}$ to the desired projection data $p \in {\mathbb{R}^{N \times N \times {N_{\text{E}}}}}$, where $N$ and ${N_{\text{E}}}$ represent patch size and the number of energy bins (energy channels), respectively (Li *et al* 2020). This process involves progressively refining $G$ in a data-driven manner to closely align the distribution of distorted spectral projections with that of the ideal data and capture the true spectral and characteristics of PCD ground-truth images. Patch training, which was also employed by Li *et al* (2020), focuses the network on a limited area, making it ideal for correcting PCD data by ensuring that cross-talk primarily occurs between neighboring pixels. Additionally, this method saves memory space and reduces the need for a large training dataset, as the network processes patches instead of entire volumetric images. This approach effectively increases the amount of equivalent data while optimizing computational efficiency (Li *et al* 2020). It is worth to mention two different key points from our previous work: changing the generator to one single network to exclude the intermediate data, and replacing the discriminator with a residual-based discriminator. This configuration enables the discriminator to efficiently differentiate between real and fake data by leveraging residual blocks, which enhances learning by mitigating the vanishing gradient problem. Additionally, this architecture takes advantage of both global and local information in the generated output, thereby providing a more comprehensive feedback signal to the generator throughout the training process. Figure 2 illustrates the network structure of both the generator and residual-based discriminator in detail.

**Generator:** inspired by the success of WGAN-VGG in previous works, here we take advantage of that considering significant improvement to directly remove pulse pileup and charge splitting in one step. Specifically, we consider one single unique network as the generator part with residual and skip connections. It is inspired by the generator and discriminator structures reported in (Li *et al* 2020) and (Yang *et al* 2018). The comprehensive setup consists of one generator and one discriminator, constructed primarily using convolutional layers and residual blocks. The generator in our R-WGAN-ViT architecture is a fully convolutional network optimized for strong representation and noise suppression. It processes input data through a series of convolutional layers that progressively reduce and then expand feature dimensions, leveraging both leaky ReLU and ReLU activations. The network employs a lightweight topology with skip connections and concatenations to facilitate training and enhance feature preservation and integration. Transposed convolutional layers are used for upsampling, with the final output produced by adding the processed data back to the input. This combination of operations effectively captures both local and global data structures, making it highly effective for deconvolution and denoising tasks. Additionally, the generator is specifically designed to address inherent features of pulse pileup and charge splitting, ensuring optimal performance by removing these distortions. Unlike the approach proposed in (Li *et al* 2020), our method eliminates the need for intermediate data, such as PCCT data after pulse pileup and Poisson noise suppression, which is often unavailable in real clinical settings.

**Residual-based discriminator:** the discriminator network is designed with residual blocks to focus on both global and local features of the generated output and to help maintain gradient flow during backpropagation, mitigating the vanishing gradient problem and enabling the training of deeper networks and still achieves compelling performance (Cai *et al* 2020). In more detail, the discriminator consists of 4 residual blocks, it begins with an initial convolutional layer that applies a convolution with 64 filters and a kernel size of 3 to the input tensor, followed by a Leaky ReLU activation function. This is followed by four residual blocks applied sequentially, each with 64, 128, 256 and 512 numbers of kernels, respectively with the identical kernel size of 3 × 3 and a stride of 2. After the residual blocks, the output is flattened and passed through a dense layer with 1024 units, where a Leaky ReLU activation function. Finally, another dense layer with a single unit is applied to produce the final output. Consequently, the discriminator becomes more adept at distinguishing between real and fake data, providing more accurate and valuable feedback to the generator. This feedback is crucial for refining the generator’s output and achieving higher-quality data generation. Residual blocks are critical components of deep neural networks, particularly in architectures such as Residual Networks (ResNet). They effectively address the issue of vanishing gradients, which can significantly hinder the training of very deep networks. The core concept of residual blocks involves the use of shortcut connections that allow gradients to bypass one or more layers, thereby facilitating more efficient learning. These shortcut connections preserve information from the input, simplifying the network’s ability to learn the identity function. This is crucial for the effective preservation of global features across multiple layers, enhancing the overall learning process (He *et al* 2016). Additionally, these connections help maintain gradient flow during backpropagation, mitigating the vanishing gradient problem and enabling the training of deeper networks. Moreover, residual connections promote a diverse range of representations within the network, enhancing its capacity to capture complex features. They enable the effective integration of contextual and convolutional features, facilitating efficient aggregation of information and ensuring that essential features are retained throughout the network (Dalmaz *et al* 2022, Imak *et al* 2023). Figure 2(c) illustrates the overall structure of a residual block extracted from figure 2(b). The residual block comprises two 3 × 3 convolutional layers with the same number of output channels. Each convolutional layer is followed by a batch normalization layer and a ReLU activation function. The unique aspect of the residual block design involves bypassing these two convolution operations and adding the input directly before the final ReLU activation function. This skip connection preserves the input and combines it with the output of the convolutional layers, enhancing feature retention. If there is a need to change the number of channels, an additional 1 × 1 convolutional layer is introduced to transform the input into the desired shape, ensuring compatibility for the addition operation. This design allows the network to maintain the integrity of the input features while applying complex transformations, thereby improving the learning efficiency and performance of the network.

#### 2.2.2. Perceptual loss (ViT)

The WGAN framework encourages the generator to reduce noise in the data distribution using a specialized loss function. Additionally, a component is incorporated into the loss function to ensure the preservation of image details and information content. This is typically achieved with a mean squared error (MSE) loss function, which aims to minimize the discrepancy between a denoised patch generated by the network and the noise-free CT image patch in a pixel-wise manner (Chen *et al* 2017). However, relying solely on MSE loss can result in images with suboptimal quality and loss of detailed information (Yang *et al* 2018). To address this issue, we employ a perceptual loss function that operates in the feature space rather than the pixel space which was proposed in pervious researches (Yang *et al* 2018, Li *et al* 2020). Our implementation leverages popular pre-trained CNNs such as VGG11, VGG13, VGG16, VGG19, Xception, and ResNet-50 as feature extractors which were also reported in our previous work (Morovati *et al* 2024). Additionally, we utilize the ViT proposed in 2020 by Dosovitskiy *et al* to extract features for the role of perceptual loss, enabling the capture of long-range dependencies between different parts of the image (Dosovitskiy *et al* 2020). ViT divides images into distinct and non-overlapping patches, and uses a transformer framework to independently analyze each patch. A notable aspect of ViT is its use of linear projection to reduce the dimensionality of these patches before introducing them to the transformer network. While reducing data in these patches might seem counterintuitive, this linear projection is essential to the ViT structure, offering significant computational efficiency (Dosovitskiy *et al* 2020, Katar and Yildirim 2023).

ViT’s design includes the self-attention mechanism, which allows the model to weigh the importance of different parts of the input image, focusing on the most relevant regions. This capability is crucial for capturing global context and intricate spatial relationships within the image, which are often missed by traditional CNNs due to their limited receptive fields. By considering long-range dependencies, ViT can create more comprehensive and detailed representations, improving the quality of the denoised images. It is worth noting that pre-trained CNNs are typically trained on the ImageNet dataset, which may differ significantly from our PCCT dataset. Therefore, employing pre-trained networks may not be the most effective approach for our specific task. To overcome this challenge, we fine-tune CNNs from scratch using our simulated PCCT dataset. These newly trained CNNs are then utilized as the perceptual loss within the R-WGAN framework. Additionally, to extract high texture features and capture long-range dependencies, we employ ViT as the feature extractor and compare the performance of R-WGAN-CNN and R-WGAN-ViT. The features are obtained from the last hidden state of the ViT model. By leveraging the self-attention mechanism, ViT provides a more nuanced understanding of the image, enhancing the denoising performance and preserving essential details and textures.

#### 2.2.3. Loss functions

The loss functions for both the generator and discriminator are crucial to train the R-WGAN-ViT. Inspired by the work of Li *et al* (2020), we modify the generator loss since our generator is made of one single network instead of two subnetworks. For the discriminator, the primary component of the loss is the Wasserstein distance, which measures the difference between the distributions of real and generated data and comes from equation (1). This is combined with a gradient penalty to enforce the Lipschitz constraint, ensuring more stable training and preventing the gradients from exploding or vanishing. The generator loss consists of three components. Considering all these error components, the overall generator loss can be expressed as:

\begin{equation*}{L_G}\, = \,\mathbb{E}\left[ {D\left( {G\left( m \right)} \right)} \right]\, + \,{\lambda _1}{\mathbb{E}_{m,p}}{\left| {G\left( m \right) - p} \right|^2}\, + \,{\lambda _2}{\mathbb{E}_{m,p}}\left| {\frac{{G\left( m \right) - p}}{{p + \in }}} \right|\, + \,{\lambda _3}\left( {{\text{Perceptual Loss}}} \right),\end{equation*}

where the primary term is the adversarial loss, which encourages the generator to produce data that the discriminator cannot distinguish from real data. Additionally, the generator loss includes the MSE term and a relative mean absolute error (RMAE) term, both of which aim to minimize the difference between the generated output and the ground truth images with the constant $\in $ in the second term fixed at 1 × ${10^{ - 4}}$ to ensure stability of the ratio. To further enhance the quality of the generated images, a perceptual loss introduced in section 2.2.2 is included to focus on high-level feature similarities rather than just pixel-wise ones. This term helps in capturing high-level features and improving the visual fidelity of the generated images. These combined loss terms, with their respective balancing weights ${\lambda _1}$, ${\lambda _2}$ and ${\lambda _3}$ guide the generator to not only create images that are indistinguishable from real ones (ground truth) but also to capture intricate details and textures, ensuring high fidelity in the generated outputs.

### 2.3. Reconstruction

The next step after applying R-WGAN-ViT for PCCT projection correction is reconstruction from the sinograms. We employ the FBP method on simulated phantom data across 256 slices and 9 different energy bins to transform the corrected data into the image domain. Each energy bin is reconstructed separately to facilitate material decomposition and other specialized applications. However, this reconstruction process inherently introduces noise, primarily due to photon starvation artifacts and quantum noise. To mitigate these effects, the traditional denoising methods are applied in the subsequent stage. These methods are essential for suppressing noise while preserving critical information, such as edges and fine details, thereby enhancing the overall quality of the reconstructed PCCT images. This dual approach ensures that the final images are both diagnostically accurate and of high quality, maintaining the integrity of important features. Additionally, for the real dataset, we apply the SART with 200 iterations to reconstruct channel images from the sinograms across different energy bins. This iterative reconstruction technique helps to further improve image quality by reducing artifacts and enhancing the visualization of key structures.

### 2.4. TV minimization denoising and GF in the image domain

#### 2.4.1. TV minimization

In this work, we employ the TV minimization denoising in the image domain to suppress the quantum noise caused by reconstruction of each energy bin due to the non-ideal response of the PCD in each energy channel. TV denoising is widely used in image processing for reducing noise while preserving important structural details such as edges. Introduced by Rudin, Osher, and Fatemi, the TV denoising algorithm works by minimizing the TV of the image, which is the sum of the absolute gradients of the image intensity (Rudin *et al* 1992). The primary objective is to find a balance between removing noise and maintaining significant image features, particularly edges, which are crucial for visual quality and interpretation. The TV denoising model can be described by the following optimization problem:

\begin{equation*}{\text{min}_u}\left\{ {\left\| {u - f\,} \right\|_2^2 + \lambda {{\left\| {\nabla u} \right\|}_1}} \right\},\end{equation*}

where $u$ is the denoised image that has smaller TV, $f$ is the observed noisy image, $\lambda $ is a regularization parameter that balances noise removal and detail preservation, and ${\left\| {\nabla u} \right\|_1}$ is the TV of the image $u$, representing the sum of the magnitudes of the gradients. In the medical imaging field, TV denoising has been extensively applied due to its effectiveness in preserving important diagnostic details while reducing noise. Medical images often contain subtle features and textures that are critical for accurate diagnosis, and TV denoising helps maintain these features by preserving edges and fine structures. For instance, TV denoising has been successfully utilized in CT to enhance image quality by reducing quantum noise and artifacts, which are common in low-dose imaging. In magnetic resonance imaging (MRI), TV denoising helps in maintaining the clarity of anatomical structures while suppressing noise, thereby improving the overall diagnostic quality of the images. A study demonstrated the application of TV denoising in LDCT, showing significant improvements in image quality by effectively reducing noise while preserving edges and important details (Sidky and Pan 2008). Another study highlighted the advantages of using TV denoising in various medical imaging modalities, emphasizing its role in maintaining the integrity of critical structures (Chambolle 2004).

In our proposed approach to solve this optimization problem, we use the TV-L1 denoising method optimized with a primal-dual algorithm. The primal-dual algorithm iteratively updates the image to reduce noise while preserving edges, balancing between data fidelity and regularization. While the general TV denoising optimization problem aims to minimize the TV of the image, the TV-L1 denoising method with a primal-dual algorithm provides a practical and effective way to achieve this by iteratively adjusting the image to reduce noise and preserve important features. Additionally, this approach is cost-effective compared to deep learning methods, which often require significant computational resources and large datasets for training (Zhang *et al* 2018). The TV-L1 method, by contrast, is lightweight, efficient, and easy to implement, making it highly suitable for real-world medical imaging applications where computational efficiency and reliability are crucial. It achieves excellent results without the need for extensive model training, offering a reliable alternative for image denoising in clinical settings.

#### 2.4.2. GF

GF is an edge-preserving technique introduced by He *et al* (2012). Unlike other traditional filtering methods that may blur edges and fine details, the GF utilizes a guidance image to preserve the structure while removing noise. The guidance image can be the input image itself or another image that shares similar structures with the input image. The guided filter is based on a local linear model, where the filtering output is locally a linear transform of the guidance image. The basic idea is to compute the filter output $q$ from the input image $p$ and the guidance image $I$ by minimizing the following cost function:

\begin{equation*}E\left( {{a_k},\,{b_k}} \right) = \,\mathop \sum \limits_{i \in {\omega _k}} \left( {{{\left( {{I_i}{a_k} + {b_k} - {p_i}} \right)}^2} + \in a_k^2} \right),\end{equation*}

where ${\omega _k}$ is a window centered at pixel $k$, ${a_k}$ and ${b_k}$ are the linear coefficients in the local window, $\in $ is a regularization parameter to avoid division by zero and the filter output ${q_i}$ is then obtained by averaging the local linear model, where $\left| \omega \right|$ is the number of pixels:

\begin{equation*}{q_i} = \frac{1}{{\left| \omega \right|}}\mathop \sum \limits_{k:i \in {\omega _k}} \left( {{a_k}{I_i} + {b_k}} \right).\end{equation*}

GF is computationally efficient and can handle high-dimensional data, making it suitable for various applications such as noise reduction, detail enhancement, and image fusion (He *et al* 2012). In the field of medical imaging, GF is particularly valuable for its ability to enhance image quality while preserving essential anatomical structures and edges. Medical images often contain intricate details critical for accurate diagnosis, and GF effectively maintains these details while reducing noise and artifacts (Diwakar *et al* 2024). In this study, we apply GF following TV denoising to further refine the denoising process. Specifically, we sum the pre-log counts from all energy bins before projection correction for both simulated and real datasets to create a virtual ‘integrating’ bin that minimizes quantum uncertainty and then apply TV denoising to reduce residual noise and artifacts in the guidance image. This virtual bin is used to reconstruct an image with significantly reduced noise, which serves as the guidance image for the GF process. By considering all the energy bins, we achieve better contrast and reduced noise, thereby preserving more details of tissues and organs. Full-energy images, which are attenuation coefficient images reconstructed using broad-spectrum projections from all available photons, usually have higher quality than single-energy images. These full-energy images are used as guidance to retain more details that might be lost in single bin noisy images. This approach ensures that the final images are of high quality, with enhanced clarity and preserved critical diagnostic information, making it an effective solution for improving medical imaging outcomes. In our approach, the guidance image is not a one-size-fits-all solution but is tailored to each body part to reflect its unique anatomical features. For each specific region, we need to use the image reconstructed from the virtual energy integration projections. That is, we can add all the pre-log photons from all the channels together to reconstruct a better image using FBP or SART.

## 3. Experiments and results

### 3.1. Dataset

#### 3.1.1. Simulated phantom dataset

To train the proposed model on PCCT data, we used the same simulated PCCT data by Li *et al* (2020). Initially, an ideal spectrum of an attenuated x-ray beam is derived from the synthesized material phantom (Poludniowski *et al* 2009). Two key components for generating PCD data are a polychromatic source and energy-dependent attenuation curves. The x-ray source spectrum was simulated using SpekCalc within a diagnostic energy range (Poludniowski *et al* 2009). The simulated spectrum spans from 12 to 120 keV with a resolution of 1 keV. The distance between the phantom and the source was set to 1.0 m, with default filtration including 0.8 mm of Beryllium, 1.0 mm of Aluminum, and 0.11 mm of Copper. For representative objects to be scanned, a group of 3D Shepp–Logan phantoms with random shapes and multiple material compositions were utilized for data generation. Each phantom was a 256 × 256 $ \times $256 cube with a voxel size of 0.113 mm^3^, containing five ellipsoids of different materials fully enclosed within a sphere of radius 12.8 mm centered in the cube. The material types assigned to the ellipsoids included soft tissue, adipose tissue, brain gray matter, white matter, blood, and cortical bone. The ellipsoids could overlap, and the material composition of an overlapped voxel was assigned to an equal-volume mixture of the involved materials. Water filled the gaps between the ellipsoids and the sphere boundary, while the space outside the sphere was treated as air.

Using the x-ray spectrum and material phantoms, 180 spectral projections were generated for each phantom in a parallel beam configuration for convenience and generality. Charge splitting effects inside the PCD were simulated using the Photon Counting Toolkit (PcTK version 3.2) developed by Taguchi *et al* (2016, 2018). This model considers different major interactions between x-ray photons within the diagnostic energy range and the PCD sensor crystal (cadmium telluride, CdTe) within a probabilistic framework, including free penetration with no detection, full detection without fluorescence, partial detection with fluorescence lost, and full detection with fluorescence reabsorbed. Compton and Rayleigh scattering were neglected due to their small probability and negligible impacts. The model assumes that one photon undergoes only one phenomenon, a simplification validated by Monte Carlo simulation. Poisson noise was added on a pixel-by-pixel basis.

The noisy, charge-splitting distorted spectral projection images were then processed through the pulse pileup model to generate the final PCD detection images. The charge splitting effect determines the distribution of charge clouds on the pixel grids, while pulse pileup arises during the readout process of these charge clouds. Poisson noise is inherently correlated due to the charge splitting effects. However, since noise correlation between pixels is not a primary concern and each pixel behaves in a Poisson manner, Poisson noise was added after the charge splitting process to achieve equivalent observations before pulse pileup. The paralyzable model for the pulse pileup effect was developed based on the work by Bierme and Roessl (2012), Roessl *et al* (2016), incorporating modifications to account for spatial cross-talk between adjacent pixels receiving different spectra, as PcTK does not include this effect. Given that the spectral resolution of the current PCD is not better than 5 keV, thresholds with a step size significantly larger than 1 keV were selected to reduce data volume. The reported energy thresholds were 20, 30, 40, 50, 60, 70, 80, 90, 100, and 110 keV. As a result, nine energy bins correspond to the ranges 20–29, 30–39, 40–49 keV, and so on, up to 100–109 keV.

**Figure 3.**
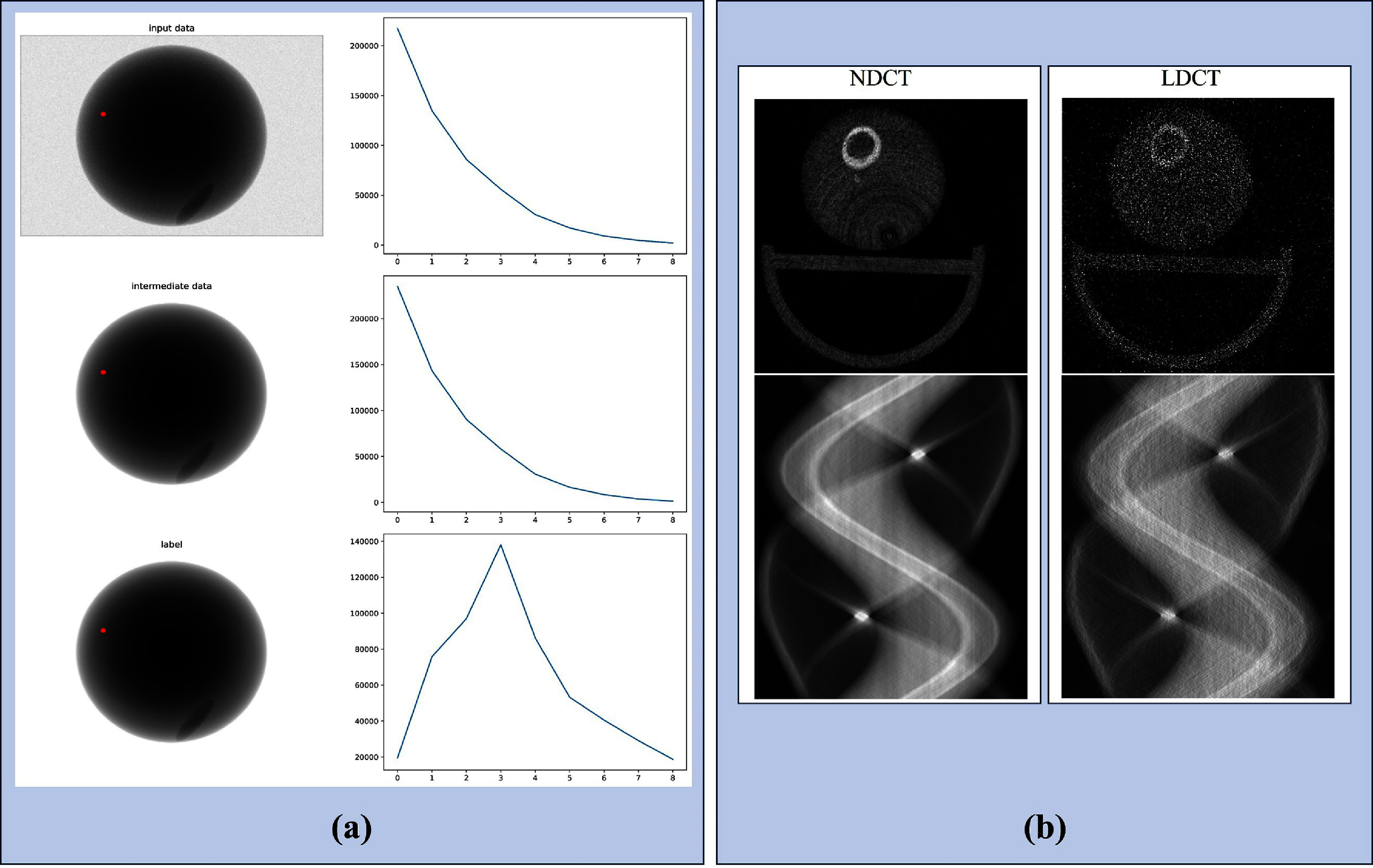
Representative projection. (a) Projection views at a 60° angle in energy bin 7 of a phantom study. From top to bottom: projections after distortions from charge splitting, Poisson noise, and pulse pileup; projections after charge splitting only; and the ground truth. The right column displays the spectral counts profile across all energy bins corresponding to the red dot indicated in the projections. (b) Reconstructions and sinograms for chicken leg data for 5th energy bin.

Experiments were carried out on 10 sets of PCD data generated from 10 randomly created 3D material phantoms, as previously described in Li *et al* (2020). Each dataset consisted of 180 spectral projections, each measuring 256 $ \times $ 367 $ \times $125, captured from various rotation angles. Another 5 material phantoms and the corresponding PCD datasets were generated for testing. Using the 10 selected thresholds detailed earlier and reported in Li *et al* (2020), the counts-above-thresholds data was converted into counts-in-bins data, thereby reducing the channel size from 125 to 9 dimensions. The 10 PCD datasets (180 $ \times $ 256 $ \times $ 367 $ \times $ 9) were then randomly divided into patches along the height and width dimensions for network training, with each patch sized at 16 $ \times $ 16 $ \times $9, to prevent GPU memory overload (Li *et al* 2020). Figure 3(a) illustrates projection views at a 60° angle in energy bin 7 and the corresponding detected counts.

#### 3.1.2. Real dataset

To evaluate the performance of our proposed method on real PCCT data, we used a scanned chicken leg phantom introduced by Niu *et al* (2022). The scans were performed using a commercial photon-counting micro-CT scanner (MARS, MARS Bioimaging Ltd, Christchurch, New Zealand) with a cone-beam circular scanning geometry. The x-ray source operated at 80 kVp/50 *μ*A, using a 1.96 mm aluminum filter. The setup included three PCD chips arranged side by side, each comprising 128 × 128 pixels. These chips used five effective energy bins in $2 \times 2$ charge summing mode, with a pixel size of 0.11 × 0.11 mm^2^. To cover the 40 mm field of view in diameter, two lateral translations were performed during the scan, and 1440 views were collected for each rotation at each translation. The energy bins were set at >7, >20, >30, >47, and >73 keV. With appropriate post-processing steps, we can convert those energy bins to non-overlapped energy ranges if the original pre-log raw projections are available. Here, the energy bins were not converted, and overlapped bins were used. For the quantitative experiments, a chicken leg with bones was placed in a plastic tube and scanned consecutively at both normal dose (300 ms exposure per view) and low dose (100 ms exposure per view) settings. Due to a significant number of defective pixels, we employed an iterative reconstruction method, specifically the SART. The reconstructions were performed with 0.1 mm isotropic voxels, using the SART algorithm for 200 iterations. The resulting volume was 550 × 550 × 130 for each energy bin, across a total of five bins. Figure 3(b) shows the reconstructions and the corresponding sinograms for 5th energy bin in both normal-dose CT (NDCT) and LDCT. Moreover, to obtain a clear sinogram, we applied several preprocessing steps. This involved removing bad pixels, combining two lateral translations, and performing subtraction on the energy bins based on the specified ranges described earlier. These steps were crucial in enhancing the quality of the final sinogram.

### 3.2. Experimental settings

We conducted our experiments on an Ubuntu 22.04. LTS system featuring an Intel (R) Core (TM) i9-9920X CPU @ 3.50 GHz. All models were implemented using TensorFlow 2.10.0 and CUDA 12.2.0, utilizing two NVIDIA RTX 6000 Ada GPUs to accelerate computations. Additionally, we used MATLAB 2023 for the traditional filtering methods. The experiment settings are as follows: The patch size is 16 as the input data and the training is optimized using Adam optimizer with training parameters as $\alpha $ $ = \,1.0\, \times \,{10^{ - 4}}$, ${\beta _1}$ $ = \,0.9$ and ${\beta _2}$ $ = \,0.999$. The hyper-parameters ${\lambda _1}$ and ${\lambda _2}$ were all chosen as 1000 and ${\lambda _3}$ was chosen as 10. For the image domain filtering, the window size or radius of GF and $\in $ were set to 5 and $1.0 \times {10^{ - 8}}$, respectively. The number of iterations for guiding was set as 1,000 iterations. For TV denoising method, $\lambda $ was chosen as 1.0 and the number of iterations was set to 18. The MSE cost converged after 40 epochs for the R-WGAN-ViT network. In this study, the simulated dataset served as the primary source of data. 10 sets of 3D material phantoms were generated for training and validation, each containing 180 spectral projections across nine energy bins. A total of 1841 400 patches were extracted from these phantoms and were shuffled to remove the connections between pairs, among these, 95% (1 749 330 pairs) were used for training, and 5% (92 070 pairs) were allocated for validation to monitor model performance. For testing, 5 additional material phantoms and their corresponding PCD datasets were generated, ensuring a completely different dataset for evaluating the model’s generalizability to unseen data allowing for a robust assessment of model performance. The real dataset, including PCCT scans of a chicken leg phantom, was reserved exclusively for evaluation and testing purposes, allowing us to independently assess the model’s performance on real-world data without any real data influencing the training process. This approach ensures that our findings demonstrate the model’s ability to generalize effectively to real PCCT data.

### 3.3. Evaluation metrics

To evaluate the performance of the proposed method, we conduct both qualitative and quantitative assessments. Quantitative evaluation is performed using three metrics: structural similarity index measurment (SSIM), root mean square error (RMSE), and Peak SNR (PSNR). SSIM measures the similarity between two images and predicts the perceived quality of digital images and videos, ranging from −1 to 1, with values closer to 1 indicating higher similarity. RMSE quantifies the difference between the results and the ground-truths, with lower values indicating better image quality. PSNR is a widely used metric for evaluating the quality of reconstructed images, measuring the ratio between the maximum possible power of a signal and the power of corrupting noise. Higher PSNR values indicate better image quality, with less distortion and noise compared to lower values. While RMSE is used in projection domain, PSNR is employed in reconstructed image domain. Additionally, noise power spectrum (NPS) is used to compare the noise characteristics of the reconstructed PCCT images with respect to the ground-truth images. We conduct a one-way ANOVA to evaluate the statistical significance of the differences in SSIM and PSNR values among the tested methods. The *p*-values are computed, and a *p*-value < 0.05 is considered to be statistically significant.

### 3.4. Projection domain results

#### 3.4.1. Simulated phantom data

We compared the effectiveness of various CNN architectures and the ViT as feature extractors for the perceptual loss component in WGAN and R-WGAN framework. Among the CNNs tested, VGG16 outperformed other architectures, including ResNet50, Xception, VGG11, VGG13, and VGG19, in extracting high-level features crucial for our task. Although different VGG networks have a similar architecture to VGG16, the number of layers in these networks plays a significant role in their feature extraction capabilities. Newer architectures like ResNet50 and Xception, designed to address vanishing gradients, did not perform as well as VGG16 in this specific application. Additionally, the ViT module proved effective in capturing both global and local information from the input image, enabling the generator to produce more realistic images. Quantitative metrics, including RMSE and SSIM, demonstrate that ViT yields superior results as the perceptual loss component, as summarized in table 1. Visual inspection of energy channel images and relative error analysis confirm the accuracy and efficacy of our method, finding that it provided slightly better RMSE and SSIM values compared to VGG16, highlighting the potential of transformers for feature extraction. This comparison underscores the promise of ViT as a viable alternative for enhancing WGAN and R-WGAN performance in PCCT data correction. It is noteworthy that there is still room for further improvements in PCCT data correction in both projection and image domain. To address these advancements, and building on our previous work (Morovati *et al* 2024), we tackled these challenges by integrating a residual-based discriminator, a single unique generator, and traditional image domain filtering techniques, thereby further minimizing spectrum distortion and quantum noise to enhance overall performance.

**Table 1.** RMSE and SSIM comparison between WGAN and R-WAGN for the corrected data with respect to ground truth in projection domain with pre-trained feature extractor and training from scratch feature extractor. The best result is marked in bold.

	Network	RMSE$ \downarrow $	SSIM$ \uparrow $
Pre-trained feature extractor	WGAN-VGG19	0.006 03	0.768 36
	WGAN-VGG16	0.003 55	0.797 25
	WGAN-ResNet50	0.005 10	0.737 63
	WGAN-Xception	0.004 93	0.839 06
	WGAN-ViT	**0.002 35**	**0.946 43**
	R-WGAN-VGG19	0.005 57	0.796 54
	R-WGAN-VGG16	0.002 13	0.813 69
	R-WGAN-ResNet50	0.004 80	0.750 36
	R-WGAN-Xception	0.004 71	0.839 19
	R-WGAN-ViT	**0.002 19**	**0.956 97**

Training from scratch feature extractor	WGAN-VGG19	0.004 16	0.812 24
	WGAN-VGG16	0.002 61	0.886 21
	WGAN-VGG13	0.003 94	0.648 54
	WGAN-VGG11	0.003 83	0.664 46
	WGAN-ResNet50	0.004 27	0.850 58
	WGAN-Xception	0.002 83	0.860 63
	WGAN-ViT	**0.002 11**	**0.950 24**
	R-WGAN-VGG19	0.004 78	0.835 46
	R-WGAN-VGG16	0.001 23	0.889 76
	R-WGAN-VGG13	0.003 19	0.715 69
	R-WGAN-VGG11	0.003 07	0.724 62
	R-WGAN-ResNet50	0.004 20	0.873 75
	R-WGAN-Xception	0.003 01	0.881 23
	R-WGAN-ViT	**0.001 13**	**0.964 93**

**Figure 4.**
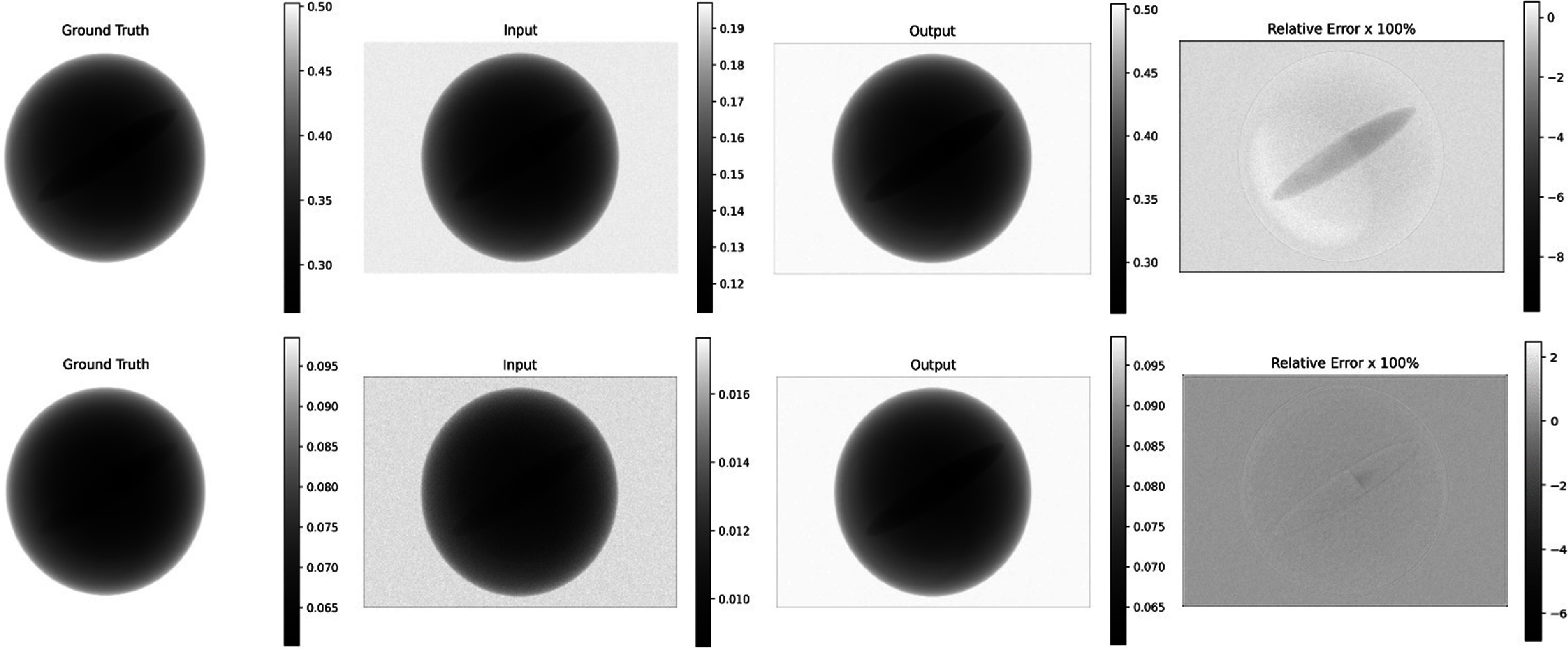
Representative projection images at $40^\circ $ angle view. The top and bottom rows are projections in energy bin 4 (50–59 KeV) and 8 (90–99 KeV), respectively. Input corresponds to the projection view before correction, and output corresponds to the projection after pulse pileup, Poisson noise and charge splitting correction. Counts are normalized with 400 000.

To evaluate the performance of the proposed R-WGAN-ViT, experiments were conducted on 10 sets of PCD data derived from 10 randomly generated 3D material phantoms, as previously utilized in Li *et al* (2020) and described in section 3.1.1. After parameter configuration and the training phase, the trained network was applied to the testing data. Figure 4 showcases two energy channel images (bin 4 and bin 8) from a representative projection view with complex structures. Visually, the corrected results are indistinguishable from the ground truths, with negligible relative errors, highlighting the effectiveness of R-WGAN-ViT. By comparing projections before and after correction, it reveals significant improvements in scale and noise level, along with a slight enhancement in structure contrast. Specifically, the scale range shifts from approximately [0.12, 0.195] before correction to [0.275, 0.5] after correction for energy bin 4, matching the ground truth range. Despite severe Poisson noise in the higher energy bin due to lower counts, the results demonstrate a clear noise reduction in energy bin 8, as shown in figure 4. For quantitative evaluation of the proposed R-WGAN-ViT, RMSE and SSIM metrics were used to compare the results considering different perceptual loss configurations and the WGAN proposed in our previous study (Morovati *et al* 2024). The results, summarized in table 1, indicate that the R-WGAN-ViT method slightly enhances image quality compared to others. Integrating residual blocks into the discriminator framework yielded improved RMSE and SSIM values compared to WGAN-ViT. Additionally, it is clear that training the CNNs or the ViT from scratch based on our PCD data can achieve better results, as reported in our previous work (Morovati *et al* 2024). Notably, even with the generator as a single unique framework and the removal of intermediate data from the correction steps, the results remained robust and slightly better, highlighting the potential of our proposed framework.

#### 3.4.2. Real data

**Figure 5.**
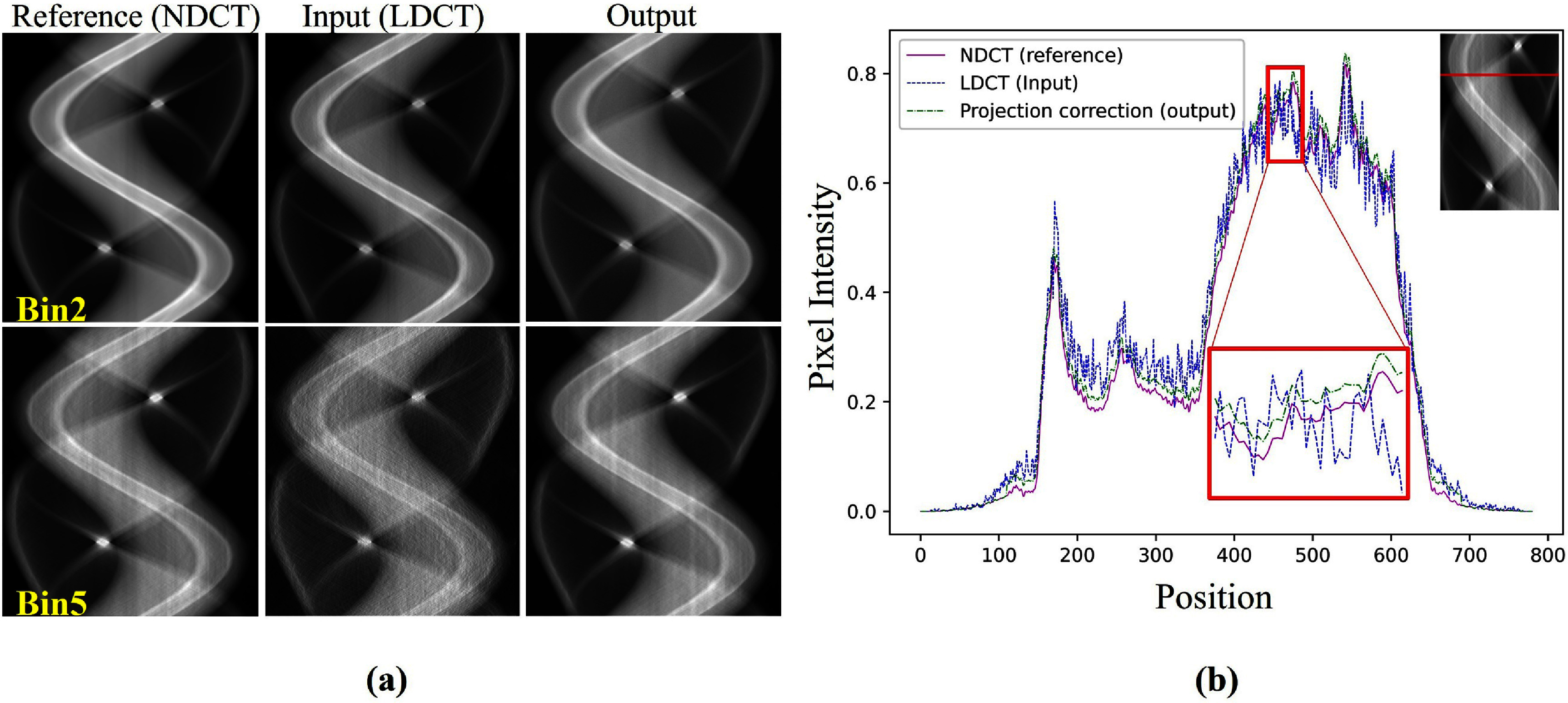
Representative sinograms and profiles of real data. (a) Shows sinogram images in energy bins 2 and 5. Input corresponds to the sinogram before correction (LDCT), and output corresponds to the sinogram after correction through R-WGAN-ViT. Images are 1440 × 781 pixels in size and the display window is [0, 0.85] ${\text{c}}{{\text{m}}^{ - 1}}$. (b) Shows the representative intensity profile of chicken leg datasets for energy bin 5 in the sinogram for the 80th image slice. The pixel position for zoomed-in view on the region of interest is from 440 to 480.

To assess the effectiveness of R-WGAN-ViT in denoising PCCT projection images, we tested the model, trained on simulated phantom data, using scans of a chicken leg captured at both low and normal dose settings, following the methodology outlined by Niu *et al* (2022). For each translation, 1440 views are acquired per rotation, and 781 detector pixels are used to cover the image diagonal across five energy bins and the low-dose images served as noisy inputs, while the normal-dose images were used as ground truth. Figure 5(a) presents qualitative results before and after applying the R-WGAN-ViT, illustrating that the corrected images closely resemble the reference images. This highlights the importance of excluding intermediate data, which is not available in this process. Figure 5(a), shows sinogram images from two energy channels (bins 2 and 5) with complex structures. The corrected results are visually indistinguishable from the ground truths, underscoring the effectiveness of R-WGAN-ViT. A comparison of projections before and after correction reveals significant improvements in scale and noise level, along with a slight enhancement in structural contrast. These results demonstrate R-WGAN-ViT’s capability to produce high-quality denoised images while preserving critical diagnostic details and reducing noise. Additionally, figure 5(b) presents the intensity profile comparison of the projection image for energy bin 5, showcasing the input (LDCT), reference (NDCT), and output of R-WGAN-ViT. As seen in the zoomed-in view, the corrected output closely follows the intensity profile of the NDCT, indicating that R-WGAN-ViT successfully reduces noise and restores the image to a quality that closely matches the normal-dose reference. The results visually affirm the model’s ability to enhance both structural accuracy and noise reduction. Quantitative metrics, including RMSE and SSIM, are summarized in table 2. Since our findings indicate that training from scratch leads to better results, we did not include a comparison using pre-trained models for perceptual loss. It is evident that, when tested on real datasets, R-WGAN-ViT outperforms other competing methods, as shown in table 2.

**Table 2.** RMSE and SSIM comparison between WGAN and R-WAGN for the corrected data with respect to reference in projection domain for the chicken leg data. The best result is marked in bold.

Network	RMSE$ \downarrow $	SSIM$ \uparrow $
WGAN-VGG19	0.004 99	0.891 15
WGAN-VGG16	0.003 11	0.917 32
WGAN-VGG13	0.004 13	0.694 14
WGAN-VGG11	0.003 86	0.712 56
WGAN-ResNet50	0.004 83	0.895 63
WGAN-Xception	0.003 42	0.906 34
WGAN-ViT	**0.002 96**	**0.926 71**

R-WGAN-VGG19	0.004 32	0.892 01
R-WGAN-VGG16	0.003 03	0.928 45
R-WGAN-VGG13	0.004 09	0.705 28
R-WGAN-VGG11	0.003 54	0.734 62
R-WGAN-ResNet50	0.004 22	0.900 22
R-WGAN-Xception	0.003 11	0.911 26
R-WGAN-ViT	**0.002 02**	**0.931 84**

### 3.5. Image domain results

#### 3.5.1. Simulated phantom data

**Figure 6.**
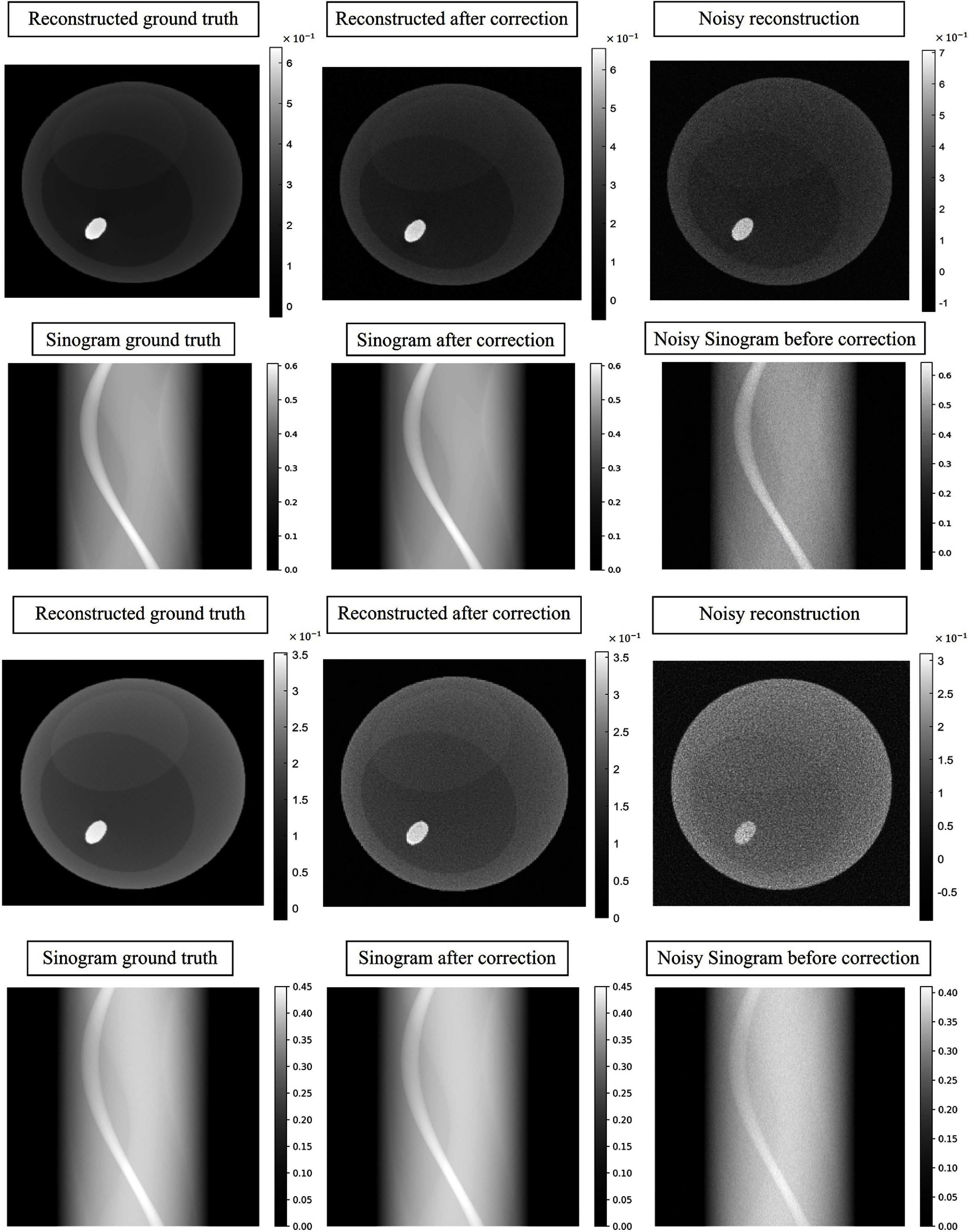
The first and second rows show the reconstructions and sinograms in bin 2 before and after correction. The third and fourth rows show the reconstructions and sinograms in bin 6 before and after correction. Reconstructed images are 256 × 256 pixels in size and sinograms are 180 × 367.

**Figure 7.**
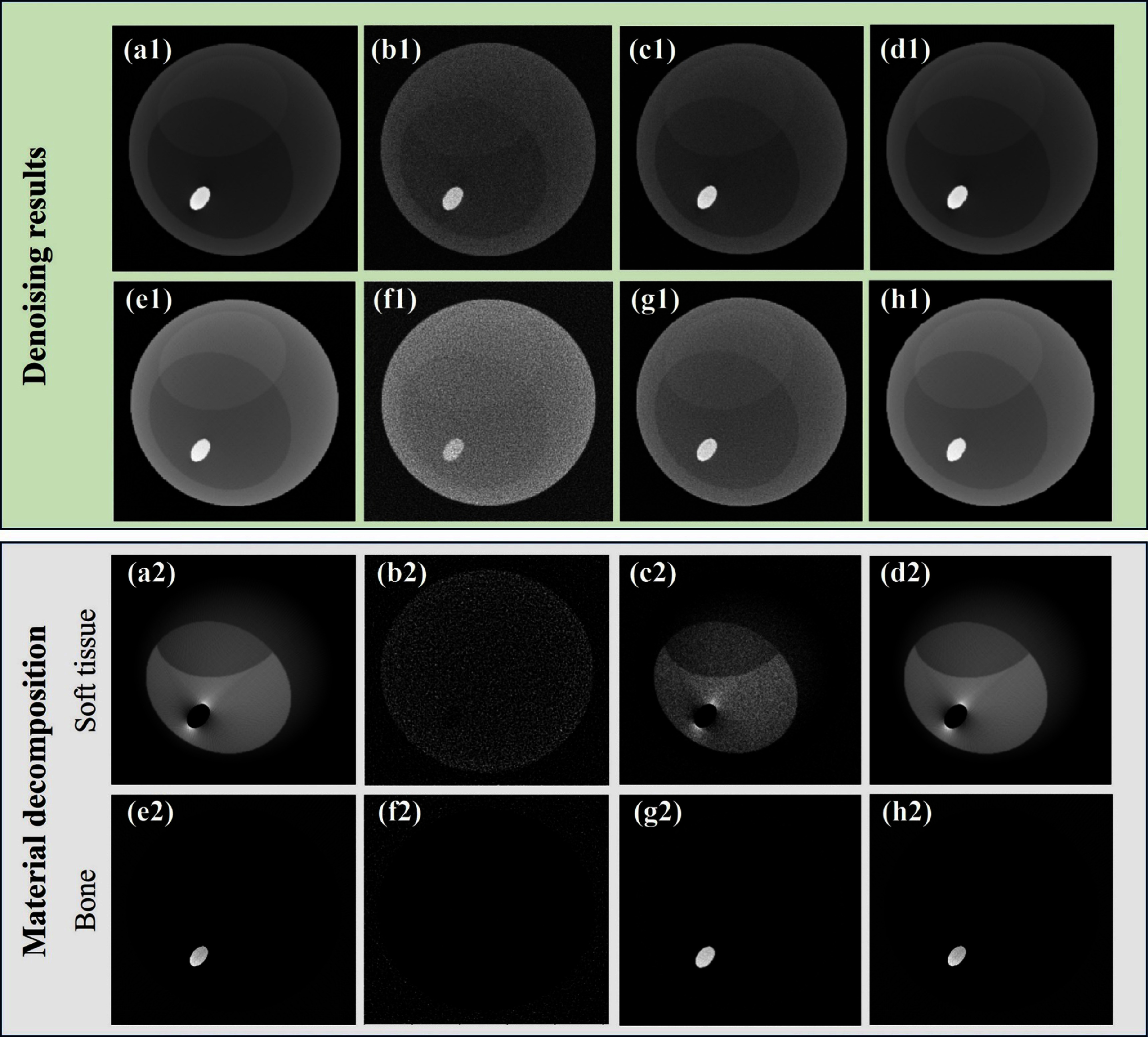
Denoising and material decomposition results of an image slice of the material phantom. The green box shows reconstructed results for different scenarios. (a1)–(d1) and (e1)–(h1) respectively show results from the 2nd and 6th energy bins. (a1) and (e1) ground truth; (b1) and (f1) reconstructed images before correction; (c1) and (g1) reconstructed results after correction using R-WGAN-ViT; (d1) and (h1) reconstructed results after correction using R-WGAN-ViT + TV + GF. The display windows range from [0, 0.65] ${\text{c}}{{\text{m}}^{ - 1}}$at the top to [0, 0.35] ${\text{c}}{{\text{m}}^{ - 1}}$ at the bottom. The gray box shows results of the material decomposition showcasing soft tissues and bone. (a2) and (e2) ground truth, (b2) and (f2) noisy data with no correction, (c2) and (g2) after projection correction using R-WGAN-ViT, and (d2) and (h2) after dual domain correction using R-WGAN-ViT + TV + GF. The display windows range from [0.01, 0.54] at the top to [0.0001, 0.008] at the bottom. Images are 256 × 256 pixels in size.

To evaluate the impact of spectral correction on reconstruction accuracy, sinograms with air correction were computed from data before and after spectral correction and subsequently reconstructed using the FBP method. Figure 6 displays the reconstructions and the corresponding sinograms of a representative slice with intricate structures (the 95th row of the projections in figure 4) for bins 2 and 6, respectively. Relying solely on projection domain corrections using R-WGAN-ViT, the reconstruction results remain noisy due to the photon count starving artifacts, and as noted by Li *et al* (2020). To effectively suppress noise while preserving edge information, we applied the TV-L1 denoising method using a primal-dual algorithm, followed by GF to further refine the denoising process using the reference image. It is important to note that the noise level varies across different energy channels. While the low energy channel experiences heavier photon absorption, low photon counts in the high energy channel result in more severe noise. As illustrated in the green box of figure 7, our proposed method, R-WGAN-ViT + TV + GF, significantly reduces noise without sacrificing critical information, which is essential for accurate diagnosis. This demonstrates the effectiveness of the dual-domain correction steps. Moreover, substantial noise reduction and contrast enhancement are evident. In particular, the second row in the green box of figure 7, corresponding to energy bin 6, which suffers from more severe noise due to lower photon counts in higher energy bins, shows a successful recovery of structures previously obscured by noise and a strong suppression of noise in the air region. The corrected results closely match the ground truths in terms of structural integrity.

Additionally, we evaluated another common filtering approach in CT denoising, specifically wavelet transform (WT) followed by GF. The results indicate that the TV-L1 method outperforms the wavelet transform in terms of noise suppression and edge preservation. For quantitative assessment, SSIM and PSNR metrics were evaluated on the reconstructed images with reference to the ground truths, and listed in table 3 in section 3.6. Not surprisingly, the quantitative results after quantum noise suppression are significantly higher than those before correction. Furthermore, the results of TV-L1 outperformed wavelet transform in terms of SSIM and PSNR, consistent with the visual impressions illustrated in figure 7.

**Table 3.** Quantitative comparison ressults using different methods with respect to ground truth for the simulated data. The best result is marked in bold. The $p - {\text{valu}}{{\text{e}}_{{\text{ANOVA}}}}$ = 0.0008 for SSIM and 0.002 for PSNR.

Methods	SSIM$ \uparrow $		PSNR$ \uparrow $	
	Reconstructed images (channel number)		Reconstructed images (channel number)	
	2nd	6th	2nd	6th
WGAN-VGG19 (Li *et al* 2020)	0.8796	0.8676	34.20	35.75
WGAN-ViT (Morovati *et al* 2024)	0.8899	0.8779	35.01	35.81
R-WGAN-ViT + Noise2Sim (Niu *et al* 2022)	0.9299	0.9175	36.97	36.81
R-WGAN-ViT + WT + GF	0.9083	0.8881	37.62	37.43
R-WGAN-ViT + TV + GF	**0.9770**	**0.9684**	**40.81**	**39.77**

Building upon these findings, we explored the impacts of our corrections in material decomposition. Material decomposition is a critical application in PCCT, enabling the differentiation of various materials and tissues based on their spectral signatures. It plays a vital role in applications such as detecting specific contrast agents, quantifying tissue composition, and improving diagnostic accuracy in clinical imaging scenarios (Flohr *et al* 2020, Flohr and Schmidt 2023). Material decomposition results reveal a significant improvement in image quality following the proposed correction method, as illustrated in the gray box of figure 7. Notably, the corrected images exhibit enhanced contrast and clarity in soft tissue and bone structures, which is essential for accurate spectral analysis and diagnostic interpretation in clinical PCCT for extremity imaging. By addressing artifacts such as charge splitting and pulse pileup, our dual-domain correction approach maintains spectral fidelity and improves the visualization of material-specific features. This enhancement is particularly valuable for applications requiring precise tissue differentiation and material decomposition, underscoring the potential clinical impact of our method. To provide a more objective evaluation of these findings, we present quantitative evaluation results for the decomposed materials. SSIM and PSNR were calculated before and after correction. The noisy input data yielded an SSIM of 0.68 with a PSNR of 20.65 for soft tissue, and an SSIM of 0.59 with a PSNR of 21.77 for bony structures. After correction using the R-WGAN-ViT model, the SSIM values improved to 0.78 for soft tissue and 0.81 for bony structures, with the corresponding PSNR values of 32.83 and 30.11 dB. Further improvements were observed with the R-WGAN-ViT+TV+GF framework, which achieved SSIM values of 0.9308 for soft tissue and 0.9178 for bony structures, along with PSNR values of 38.99 and 36.97 dB, respectively. The results indicate a significant improvement in both SSIM and PSNR values after applying our method, further validating the effectiveness of the proposed framework.

#### 3.5.2. Real data

**Figure 8.**
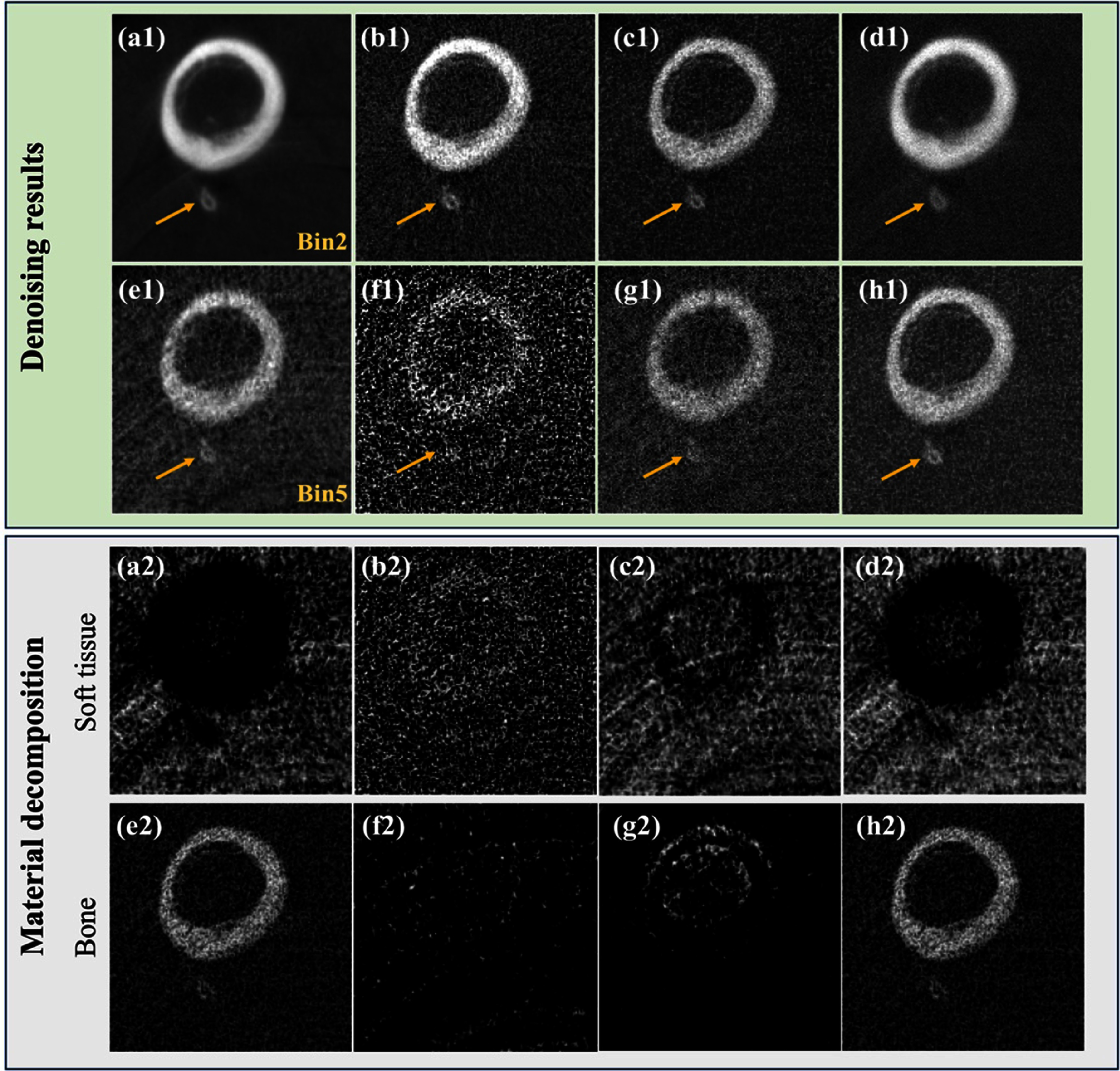
Denoising and material decomposition results of an image slice of the real chicken leg phantom. The green box shows reconstructed results for different scenarios. (a1)–(d1) and (e1)–(h1) show results from the 2nd and 5th energy bins, respectively. (a1) and (e1) Reference (NDCT-300 ms); (b1) and (f1) results before correction (LDCT-100 ms); (c1) and (g1) reconstructed results after correction using R-WGAN-ViT; (d1) and (h1) reconstructed results after correction using R-WGAN-ViT + TV + GF. The display window ranges from [0, 0.75] $\text{cm}^{ - 1}$. The gray box shows results of the decomposed soft tissue and bony components. (a2) and (e2) Reference (NDCT-300 ms), (b2) and (f2) decomposed results before correction (LDCT-100 ms), (c2) and (g2) decomposed results after correction using R-WGAN-ViT, and (d2) and (h2) decomposed results after dual domain correction using R-WGAN-ViT + TV + GF. The display windows range from [0.002, 0.07] at the top to [0.0001, 0.025] at the bottom. Images are 161 × 151 pixels in size.

The model trained on the simulated phantom data was tested on a real chicken leg phantom, and re-training or transfer learning was not applied. The green box of figure 8 presents the reconstruction results for the chicken leg data before and after correction by applying the R-WGAN-ViT. The improvements in the quality of the corrected projections are evident. However, there remains a need for additional denoising in image domain. To address this, we employ a combination of TV-L1 denoising and GF, effectively suppressing quantum noise while preserving essential image details. Similarly, in this process, the full energy image serves as the reference for GF, ensuring that the final images maintain high fidelity and clarity. Since the energy bins have overlap, the full energy images in fact are reconstructed from bin 1. This dual-stage approach not only enhances the visual quality of the PCCT images but also ensures that critical diagnostic information is retained. By integrating these advanced denoising techniques, we achieve a significant reduction in noise levels and an improvement in structural contrast, making the corrected images more reliable for clinical evaluation. The results are also illustrated in the green box of figure 8 before and after denoising through TV + GF. This comprehensive method demonstrates the robust capabilities of R-WGAN-ViT in conjunction with traditional denoising methods, offering a powerful solution for improving the overall quality of PCCT imaging. We can see that the part specified by the yellow arrow is much clearer after denoising through TV + GF. Additionally, based on the results in the green box of figure 8, we can conclude that our model effectively mitigates ring artifacts, even when they present in reference or ground truth images. Material decomposition results, highlighted in the gray box of figure 8, demonstrate an enhancement in image quality following the implementation of our correction method. The corrected images show significantly improved contrast and clarity in both soft tissues and bony structures. To quantitatively evaluate these improvements, SSIM and PSNR were computed. The noisy input resulted in SSIM values of 0.61 for soft tissues and 0.56 for bony structures, with the corresponding PSNR values of 21.35 and 22.63 dB, respectively. After applying the R-WGAN-ViT correction, the SSIM values increased to 0.69 for soft tissues and 0.75 for bone structures, with PSNR values of 25.19 and 27.39 dB, respectively. Further enhancements were achieved using the R-WGAN-ViT + TV + GF framework, which yielded SSIM values of 0.9027 for soft tissues and 0.9144 for bony structures, accompanied by PSNR values of 32.58 and 33.11 dB. These results highlight the improvements in image quality, confirming the effectiveness of the proposed framework in addressing noise and artifacts while maintaining critical diagnostic information.

### 3.6. Comparison analysis

**Figure 9.**
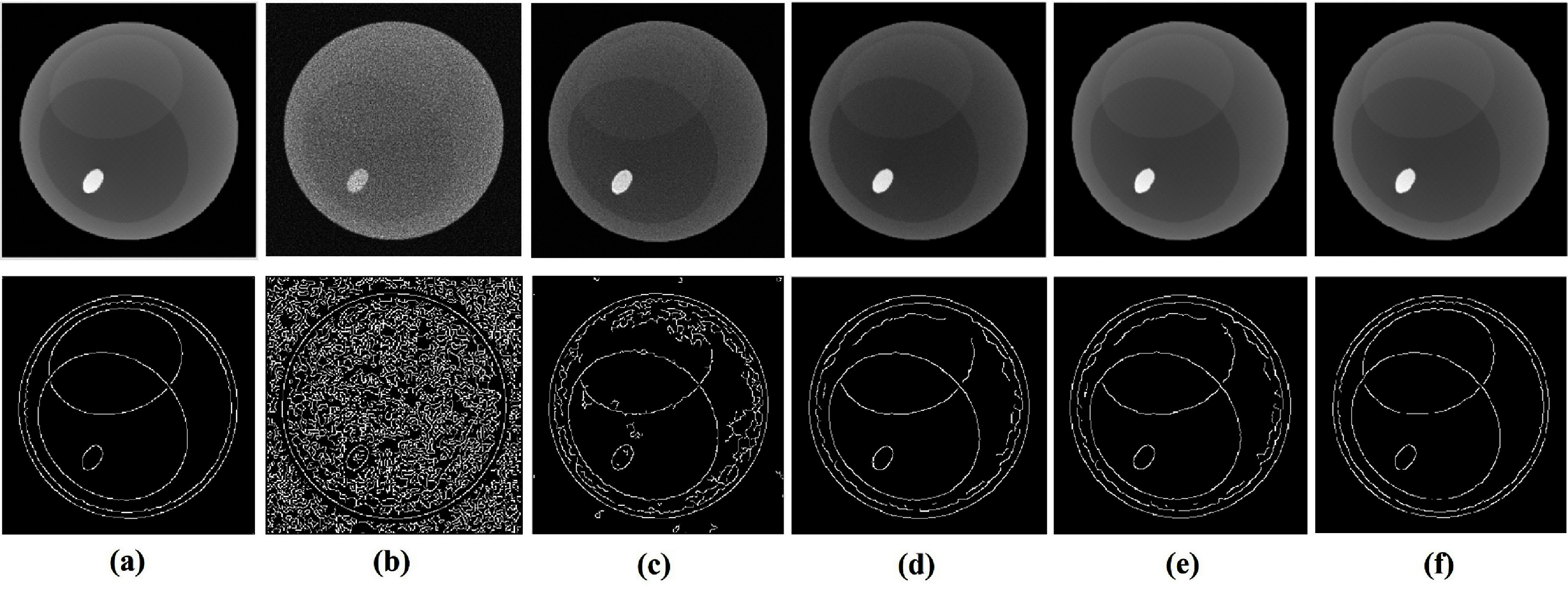
The first row shows representative images reconstructed from simulated data using different correction methods (the 95th row of the projections) in energy bin 6 and display window is [0, 0.35] ${\text{c}}{{\text{m}}^{ - 1}}$. The second row shows the results of canny edge detector on the first-row images. (a) The ground truth; (b) noisy image; (c) WGAN-VGG19; (d) R-WGAN-ViT + Noise2Sim; (e) R-WGAN-ViT + WT + GF; and (f) R-WGAN-ViT + TV + GF (ours). Images are 256 × 256 pixels in size.

To evaluate the effectiveness of our dual-domain PCCT data correction approach, we investigated and compared the following state-of-the-art models: WGAN-VGG19 (Li *et al* 2020), WGAN-ViT (Morovati *et al* 2024), and Noise2Sim (Niu *et al* 2022). Each of these methods was meticulously implemented and optimized using their respective official open-source codes. Since Noise2Sim belongs to the post-processing category, we compared it with our TV + GF denoising method. Initially, projection domain correction was applied, followed by denoising methods in the image domain. The first row of figure 9 presents representative denoising results using different methods for the simulated data. The first and second columns display the ground truth and the noisy reconstructed image, respectively. The third column illustrates the reconstruction results of WGAN-VGG19 without any subsequent image domain denoising. The remaining columns illustrate the R-WGAN-ViT framework for projection correction, followed by Noise2Sim, WT + GF, and TV + GF as post-processing noise removal methods, respectively. It is evident that the result of R-WGAN-ViT + TV + GF closely resembles the ground truth. The WGAN-VGG19 method struggles to clearly define certain structures, especially edges. In contrast, our model, R-WGAN-ViT + TV + GF, significantly suppresses noise after reconstruction while preserving structures and edges effectively. This highlights the superior performance of our proposed dual-domain correction approach.

As the aforementioned, we also evaluated wavelet transform followed by GF as a post-processing denoising method. The results indicate that TV + GF outperforms the wavelet transform and other methods in terms of noise suppression and edge preservation. For quantitative assessment, SSIM and PSNR metrics were evaluated on the reconstructed images with respect to the ground truths, as listed in table 3. The results of TV + GF score significantly higher than other methods and those without image domain denoising in both metrics. The differences are also evaluated by the ANOVA test, and the *p*-values are computed to determine whether there are statistically significant differences between different methods. Furthermore, these quantitative results are consistent with the visual impressions illustrated in figure 9.

For further investigation and showing the effectiveness of our dual-domain approach, we applied a famous edge detection algorithm on the reconstrecuted images denoised by different methods. In CT imaging, Canny edge detection is particularly useful for highlighting the boundaries of anatomical structures, aiding in tasks such as tumor detection, organ segmentation, and the precise measurement of tissue dimensions. The representative results are depicted in the second row of figure 9. We can clearly see that the edges are perfectly preserved by TV + GF, but with WT + GF and Noise2Sim there are some corrupted areas. Moreover, we can see the crucial role of post-processing denoising in PCCT data since when there is no noise suppression technique there will be residual noise caused by photon count starving artifact after reconstruction.

**Figure 10.**
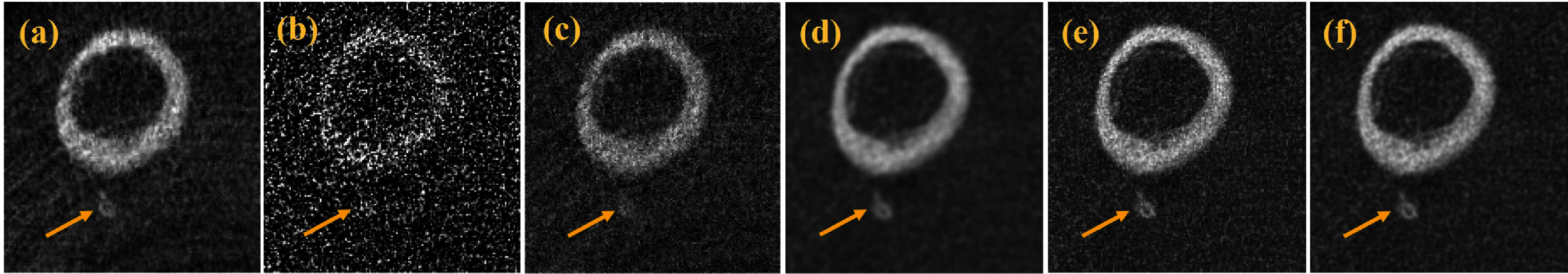
Representative images reconstructed from chicken leg data using different correction methods in energy bin 5. (a) Reference (NDCT-300 ms); (b) reconstructed image before correction (LDCT-100 ms); (c) WGAN-VGG19; (d) R-WGAN-ViT + Noise2Sim; (e) R-WGAN-ViT + WT + GF; and (f) R-WGAN-ViT + TV + GF (ours). Yellow arrows indicate a small bony structure. Images are 161 × 151 pixels in size and the display window is [0, 0.75] ${\text{c}}{{\text{m}}^{ - 1}}$.

Similarly, figure 10 shows a comparison of different methods applied to the chicken leg data. It is evident that the output of the R-WGAN-ViT + TV + GF approach closely resembles the normal-dose image, in terms of the bone structures and overall image quality. Moreover, both noise and artifacts, including ring artifacts, are significantly reduced, even though ring artifact correction is not a specific focus of this study. The WGAN-VGG19 method struggles to clearly define certain structures, especially fine details. In contrast, our model, R-WGAN-ViT + TV + GF, significantly suppresses noise after reconstruction while preserving structures and edges effectively. This highlights the superior performance of our proposed dual-domain correction approach. Moreover, the quantitative results for different methods are summarized in table 4 for energy bins 2 and 5, in terms of SSIM and PSNR. The differences are also assessed by the ANOVA test, and the *p*-values are reported to show statistically significant differences between different methods. These metrics indicate that R-WGAN-ViT + TV + GF outperforms the other methods, consistent with the visual comparisons shown in figure 10. By effectively combining projection correction with sophisticated post-processing denoising techniques, our method achieves a notable reduction in noise levels and an improvement in structural clarity. This comprehensive approach ensures that the final images maintain high diagnostic quality, underscoring the robustness and efficacy of our dual-domain PCCT data correction strategy. Moreover, we can clearly see that the PSNR and SSIM are significantly increased compared with applying Noise2Sim as the image domain denoising.

**Table 4.** Quantitative ressults comparison using different methods with respect to refrence image for chicken leg dataset. The best result is marked in bold. The $p - {\text{valu}}{{\text{e}}_{{\text{ANOVA}}}}$ = 0.001 for SSIM and 0.0004 for PSNR.

Methods	SSIM$ \uparrow $		PSNR$ \uparrow $	
	Reconstructed images (Channel number)		Reconstructed images (channel number)	
	2nd	5th	2nd	5th
WGAN-VGG19 (Li *et al* 2020)	0.7843	0.7312	32.09	31.82
WGAN-ViT (Morovati *et al* 2024)	0.7959	0.7448	32.20	31.83
R-WGAN-ViT + Noise2Sim (Niu *et al* 2022)	0.9134	0.9101	33.89	33.80
R-WGAN-ViT + WT + GF	0.9366	0.9204	32.76	32.34
R-WGAN-ViT + TV + GF	**0.9412**	**0.9325**	**34.51**	**34.11**

### 3.7. Ablation study results

In the ablation study section, we investigate the impact of various components and parameters of our proposed method, focusing on four main aspects: the effect of perceptual loss, the incorporation of residual blocks in the discriminator, the role of TV-L1 denoising, and the influence of GF on the denoising process. This examination is essential for understanding the contribution of each element to the overall system performance. The quantitative metrics, SSIM and PSNR, summarized in table 5 for simulated phantom data, underscore the critical importance of each component in PCCT data correction. The differences are also evaluated by the ANOVA test, and the *p*-values are reported. Previous studies (Yang *et al* 2018, Li *et al* 2020) have shown that relying solely on MSE loss can result in images with suboptimal quality and loss of detailed information. To address this, we employ a perceptual loss function that operates in the feature space rather than the pixel space, significantly enhancing the preservation of image details and considering long range features dependencies using ViT.

**Table 5.** Ablation study for the effect of each component in our proposed framework for dual domain PCCT data correction with respect to ground truth. The $p - {\text{valu}}{{\text{e}}_{{\text{ANOVA}}}}$ = 0.000 02 for SSIM and 0.003 for PSNR.

Methods	SSIM$ \uparrow $		PSNR$ \uparrow $	
	Reconstructed images (channel number)		Reconstructed images (channel number)	
	2nd	6th	2nd	6th
R-WGAN + TV + GF	0.8653	0.8503	37.78	38.50
WGAN-ViT + TV + GF	0.9680	0.9684	39.14	39.01
R-WGAN-ViT + GF	0.9445	0.9382	38.41	37.32
R-WGAN-ViT + TV	0.9106	0.9071	36.01	35.99
R-WGAN-ViT + TV + GF	0.9770	0.9684	40.81	39.77

**Figure 11.**
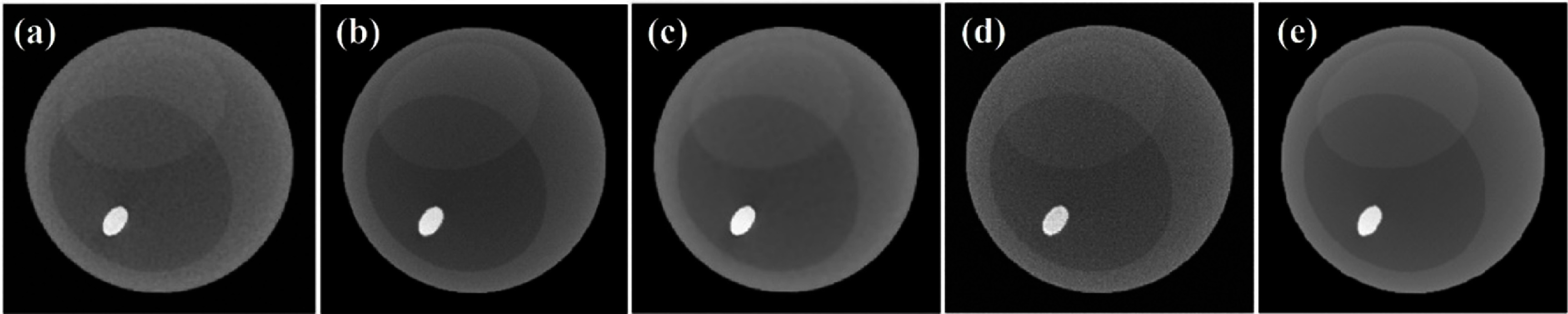
Ablation study for the effect of each component for energy bin 6. (a) R-WGAN + TV + GF, (b) WGAN-ViT + TV + GF, (c) R-WGAN-ViT + GF, (d) R-WGAN-ViT + TV and (e) R-WGAN-ViT + TV + GF. Images are 256 × 256 pixels in size and display window is [0, 0.35] ${\text{c}}{{\text{m}}^{ - {\text{1}}}}$.

Moreover, integrating residual blocks into the WGAN-ViT + TV + GF model to form the R-WGAN-ViT + TV + GF configuration allows for the consideration of both local and global information, allowing to learn residual functions and address degradation problems leading to slight improvements in performance. Introducing TV-L1 denoising to the R-WGAN-ViT + GF setup (leading to R-WGAN-ViT + TV + GF) boosts the SSIM and PSNR reducing noise and more consistent structural integrity compared to R-WGAN-ViT + GF without TV-L1. Additionally, the inclusion of GF further significantly enhances the denoising process by using the full energy image or as guidance during denoising iterations. These two components play a significant role in suppressing quantum noise, and the denoised images using TV-L1 followed by GF outperform those without these methods. Ultimately, both PSNR and SSIM metrics show remarkable improvement when the image domain denoising framework is utilized using TV + GF, underscoring its benefits. Each component (GF, TV-L1 denoising, residual blocks, and perceptual loss with ViT) contributes significantly to the overall performance of the dual-domain correction framework. The results of the ablation study can be observed in figure 11, where it illustrates the contribution of each component to the final image quality.

## 4. Discussion and conclusion

PCCT is an emerging technique with the potential to revolutionize clinical imaging. This study focused on developing and characterizing a method for dual-domain PCCT data correction. We proposed an end-to-end deep learning based approach for PCD data correction aimed at achieving high spectral fidelity. The effectiveness of our method was validated using realistically synthesized PCD data and real data, demonstrating its capability to enhance image quality and preserve essential structural details. The proposed dual-domain correction framework for PCCT data has demonstrated significant improvements in image quality through a combination of advanced neural network architectures and traditional denoising methods. Our approach integrates spectral correction in the projection domain using a R-WGAN-ViT and subsequent noise suppression in the image domain through TV-L1 denoising and GF. In our experiments, the use of ViT as a feature extractor for perceptual loss proved to be highly effective, outperforming other CNN architectures such as ResNet50, Xception, VGG11, VGG13, VGG16 and VGG19. Furthermore, the incorporation of the ViT as a feature extractor demonstrated even greater potential, achieving better RMSE and SSIM values compared to the traditional CNN-based methods. This highlights the ViT’s capability to capture long-range dependencies and its suitability for complex image reconstruction tasks.

**Figure 12.**
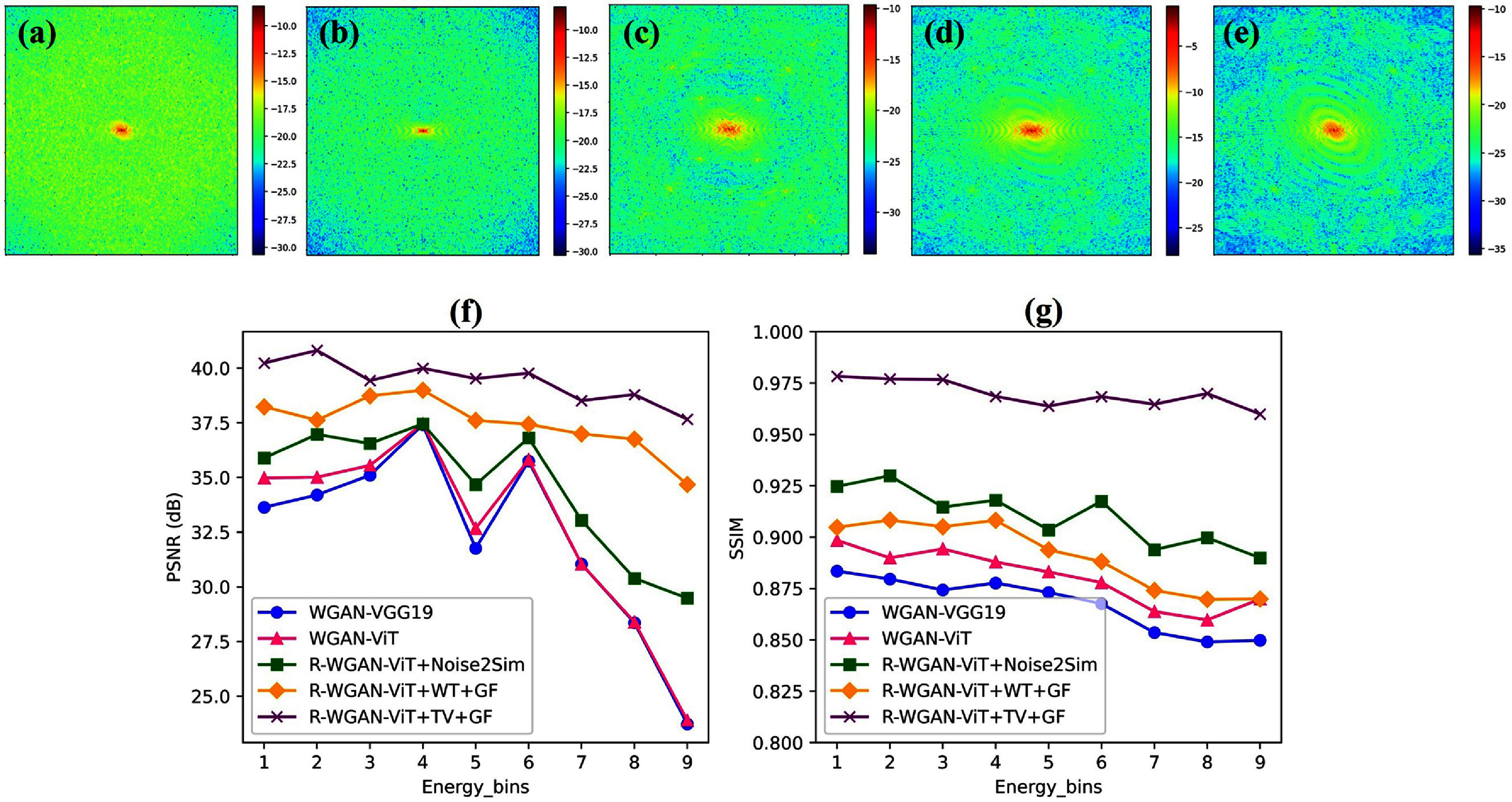
Performance analysis of different methods. (a)–(e) are comparison of the NPS maps with respect to the reference image (noise free) on the simulated data (the 95th row of the projections for energy bin 6). (a) The noisy image; (b) WGAN-VGG19; (c) R-WGAN-ViT + Noise2Sim; (d) R-WGAN-ViT + WT + GF; and (e) R-WGAN-ViT + TV + GF. (f) and (g) Show the performance analysis of different methods in terms of PSNR and SSIM values with respect to the energy bins (1–9) on the simulated data (the 95th row of the projections).

The application of TV-L1 denoising followed by GF in the image domain was crucial for preserving structural details and edges while effectively suppressing noise. This dual-domain approach addresses both pulse pileup and charge splitting distortions in the projection domain and quantum noise in the image domain. The results, validated by both visual inspections and quantitative metrics (SSIM and PSNR) for both simulated and real PCCT data, indicate that our method significantly enhances the quality of reconstructed images, making them comparable to ground truth images. Moreover, these corrections lead to more accurate attenuation values across energy bins, which directly benefit material decomposition tasks. The clearer separation of materials, improved contrast, and reduced artifacts resulting from our framework significantly enhance the reliability and diagnostic potential of material decomposition in PCCT imaging. Using simulated phantom data, figure 12 further substantiate our findings. The NPS plots in figures 12(a)–(e) illustrate the superior denoising power of our model compared to others, clearly demonstrating its effectiveness in reducing noise while maintaining image integrity. Figures 12(f) and (g) provide a quantitative comparison of SSIM and PSNR between different methods, highlighting the consistent performance improvements achieved by our approach. These figures show that our method not only reduces noise but also preserves essential structural details, resulting in higher SSIM and PSNR values compared to other state-of-the-art techniques. Regarding the computational cost, the total processing times for the framework for simulated and real data, including the R-WGAN-ViT correction and subsequent image domain denoising, are approximately 12.5 and 15.4 min for the testing phase, respectively. This efficiency makes it feasible for clinical implementation, particularly given the significant improvements in image quality observed.

The ablation studies confirmed the importance of each component in our framework. The perceptual loss function, operating in the feature space, proved essential for maintaining image quality and detail, as opposed to solely using MSE loss. The inclusion of residual blocks in the discriminator facilitated the extraction of both local and global features, contributing to overall performance improvements. TV-L1 denoising, followed by GF, played a vital role in reducing noise and preserving edge information, ultimately leading to better diagnostic accuracy. Compared to other the state-of-the-art methods, such as Noise2Sim and previous WGAN implementations, our dual-domain correction framework consistently produced superior results. The integration of advanced neural network components and traditional denoising techniques offers a robust solution for PCCT data correction, capable of handling the inherent challenges of low-dose imaging and spectral distortions. It should be pointed out that there is a strong need for extensive testing across multiple datasets and conditions for further studies, and we intend to expand our experimental framework to include different patient data and different human body parts acquired on different PCCT scanners. This aligns with recent guidelines within the radiology community, which emphasize the importance of rigorous testing and validation for AI-based methods (Kim *et al* 2019, Park *et al* 2022, 2023a, 2023b, Kunst *et al* 2024).

In conclusion, the proposed dual-domain correction framework has proven to be highly effective for PCCT data correction. The workflow involves two key stages: spectral correction in the projection domain using R-WGAN-ViT and noise suppression in the image domain through TV-L1 denoising followed by GF. Our approach successfully integrates spectral correction in the projection domain and noise suppression in the image domain, leading to significant improvements in image quality by addressing quantum noise, pulse pileup, and charge-splitting artifacts. By employing advanced neural network architectures such as ViT for perceptual loss, and utilizing TV-L1 denoising followed by GF, our method addresses both global and local image features, ensuring the preservation of critical structures and edges in the image domain while suppressing quantum noise. The experimental results, supported by visual inspections and quantitative metrics (SSIM and PSNR), confirm the superior performance of our framework compared to existing the state-of-the-art methods for both simulated phantom data and real data. Material decomposition results demonstrate enhanced contrast and clearer differentiation of tissues and materials after applying our corrections, further validating the clinical potential of our method. The NPS analysis further validates the enhanced denoising capability of our approach. PCCT, with its potential to revolutionize clinical imaging, benefits greatly from our proposed method, which ensures high spectral fidelity and improved image quality. Future research will focus on further optimizing the network architectures and exploring additional advanced techniques, such as emerging diffusion models, to continue enhancing image quality and computational efficiency. Our dual-domain correction framework sets a new potential benchmark in PCCT image reconstruction, paving the way for more accurate and reliable clinical applications.

## Data Availability

The data cannot be made publicly available upon publication because no suitable repository exists for hosting data in this field of study. The data that support the findings of this study are available upon reasonable request from the authors.
